# Sheath-tailed bats (Chiroptera: Emballonuridae) from the early Pleistocene Rackham’s Roost Site, Riversleigh World Heritage Area, and the distribution of northern Australian emballonurid species

**DOI:** 10.7717/peerj.10857

**Published:** 2021-02-25

**Authors:** Tyler R. King, Troy J. Myers, Kyle N. Armstrong, Michael Archer, Suzanne J. Hand

**Affiliations:** 1 Earth and Sustainability Science Research Centre, School of Biological, Earth and Environmental Sciences, University of New South Wales, Sydney, NSW, Australia; 2 School of Biological Sciences, The University of Adelaide, Adelaide, SA, Australia; 3 South Australian Museum, North Terrace, Adelaide, SA, Australia

**Keywords:** Sheath-tailed bats, Emballonuridae, Pleistocene, Riversleigh World Heritage Area, Australian distribution

## Abstract

Sheath-tailed bats (Family Emballonuridae) from the early Pleistocene Rackham’s Roost Site cave deposit in the Riversleigh World Heritage Area, north-western Queensland are the oldest recorded occurrence for the family in Australia. The fossil remains consist of maxillary and dentary fragments, as well as isolated teeth, but until now their precise identity has not been assessed. Our study indicates that at least three taxa are represented, and these are distinguished from other Australian emballonurids based on morphometric analysis of craniodental features. Most of the Rackham’s Roost Site emballonurid remains are referrable to the modern species *Taphozous georgianus* Thomas, 1915, but the extant species *T. troughtoni* Tate, 1952 also appears to be present, as well as a very large, as-yet undetermined species of *Saccolaimus* Temminck, 1838. We identify craniodental features that clearly distinguish *T. georgianus* from the externally very similar *T. troughtoni*. Results suggest that the distributions of *T. georgianus* and *T. troughtoni* may have overlapped in north-western Queensland since at least the early Pleistocene.

## Introduction

Living emballonurids are insectivorous bats with long narrow wings, large eyes, and a tail that protrudes through a fold of skin in the uropatagium. Globally, 54 modern sheath-tailed bat species in 14 genera are distributed throughout the Old and New World tropics and subtropics (Bonaccorso, 2019), with a fossil record extending back to the middle Eocene (~48 million years ago) of Europe (Storch, Sigé & Habersetzer, 2002; Smith et al., 2012). In Australia, two genera and eight species are currently recognised: *Taphozous australis*, *T. georgianus*, *T. hilli*, *T. kapalgensis*, *T. troughtoni*, *Saccolaimus flaviventris*, *S. mixtus* and *S. saccolaimus* (Churchill, 2009). No Australian fossil emballonurids have been described.

Australian *Taphozous* species are primarily cave-dwelling bats restricted to the northern part of the continent. The Coastal Sheath-tailed Bat, *T. australis*, of north-eastern Queensland is the only non-endemic species, being also found in New Guinea (Churchill, 2009). *Saccolaimus* species are larger bats that use both trees and caves as roosts, and all have extralimital ranges that include New Guinea. In Australia, *Saccolaimus flaviventris* has the widest range of any emballonurid, being absent only from southwestern Australia and Tasmania (Churchill, 2009).

Australian emballonurid taxonomy has been revised several times in recent decades (Chimimba & Kitchener, 1991; Reardon & Thomson, 2002; Thomson, Pavey & Reardon, 2001), with several changes made to the classification and geographic distribution of modern species. Among the most significant revisions involves the recognition, following allozyme-based analyses (Reardon & Thomson, 2002), of the surprisingly widespread distribution and abundance of *Taphozous troughtoni* (Churchill, 2009). This species was originally described by Tate (1952) on the basis of a few large *Taphozous* specimens from the Mount Isa area of north-western Queensland, but thereafter routinely misidentified as *T. georgianus* throughout Queensland. The distributions of these two externally very similar species are now recognised to be both extensive but overlapping in north-western Queensland (Churchill, 2009).

Approximately 250 km north of this overlap zone in north-western Queensland, fossil emballonurid specimens have been recovered from the cave deposit known as Rackham’s Roost Site, in the Riversleigh World Heritage Area (Archer, Hand & Godthelp, 2000). This deposit was previously estimated by biocorrelation to be early Pliocene in age (Archer, Hand & Godthelp, 2000; Hand, 1995; Hand, 1996; Godthelp, 1997), but is now interpreted to be of early Pleistocene age based on radiometric dating of speleothem in direct contact with the bone deposit (~2.1 Ma; Woodhead et al., 2016). This Riversleigh fossil assemblage mostly represents the remains of small vertebrate prey, such as frogs, birds, rodents and small marsupials, brought into the original cave by carnivorous Ghost Bats (*Macroderma gigas*; Archer, Hand & Godthelp, 2000; Hand, 1996; Boles, 1998; Klinkhamer & Godthelp, 2015; Nguyen, Hand & Archer, 2016). Also preserved are the remains of several bat species including early representatives of extant taxa (e.g. *Macroderma gigas* and the Orange Diamond-faced Bat *Rhinonicteris aurantia*; Hand, 1996) as well as extinct species (e.g. *Hipposideros winsburyorum* Hand & Godthelp, 1999 and *Megaderma richardsi* Hand, 1995). Hand (1996, 2006) noted that at least two emballonurid taxa were also represented in the Rackham’s Roost Site deposit and that these were probably species of *Taphozous*, the oldest and only fossils of this bat family so far recorded from the Australian continent (Archer, Clayton & Hand, 1984; Hand, 2006). Continued collection and acid-processing of the Rackham’s Roost Site fossiliferous limestone has now resulted in the recovery of a total of 63 craniodental emballonurid specimens. Our study focused on assessing whether these early Pleistocene emballonurid fossils are referrable to the genus *Taphozous*, whether they represent extant or extinct species or both, and whether they might include either of the extant species *T. georgianus* and *T. troughtoni* whose modern distributions overlap in this region of northern Australia.

## Materials and Methods

### Materials

The fossil emballonurid specimens described here were recovered by acetic acid processing of fossiliferous limestone collected from Rackham’s Roost Site in the Riversleigh World Heritage Area, north-western Queensland. Fossil specimens are from the Queensland Museum fossil collection, Brisbane, Australia (prefix QM F), and the University of NSW vertebrate fossil collection, Sydney, Australia (prefix AR).

Comparative material used in this study included the skulls of 90 modern emballonurids as follows: *Saccolaimus flaviventris* (seven specimens), *S. saccolaimus* (2), *Taphozous australis* (9), *T. georgianus* (55), *T. hilli* (7) and *T. troughtoni* (10) (Supplemental Datas S1 and S2). These specimens are from the American Museum of Natural History, New York, NY, USA (prefix AMNH), Australian Museum mammal collection, Sydney, Australia (prefix AM M), and the Museum and Art Gallery of the Northern Territory, Darwin, Australia (prefix U).

### Cranial and dental measurements

**Figure 1:**
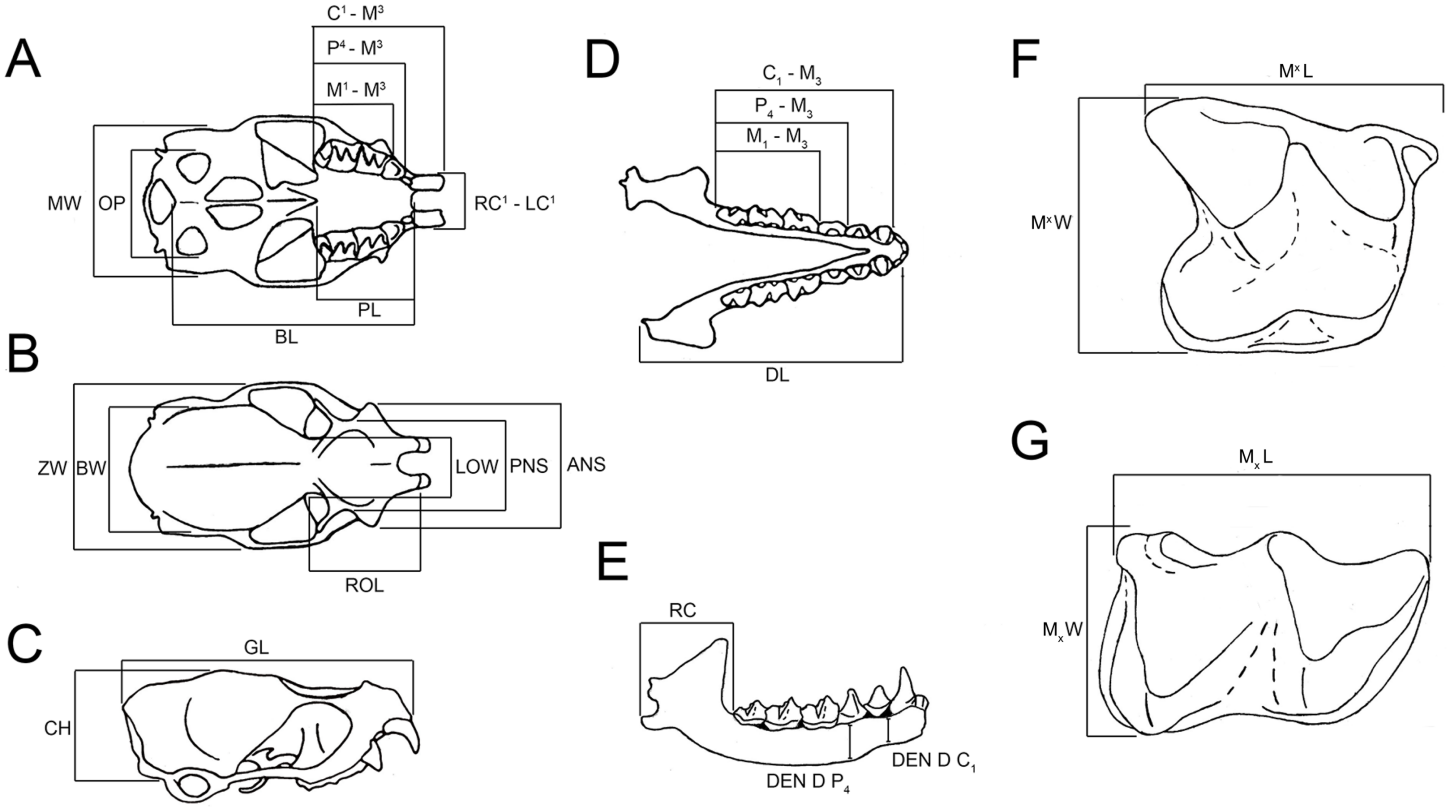
Measurements referred to in the text for skull, dentary, upper and lower molars (*Taphozous georgianus*), excluding variables excluded from multivariate analyses as uninformative. (A) Skull, ventral view; (B) skull, dorsal view; (C) skull, lateral view; (D) dentaries, dorsal view; (E) dentary, buccal view; (F) upper molar, occlusal view; (G) lower molar, occlusal view. Adapted from Chimimba & Kitchener (1991) and Hand (1985). Complete measurement list, including variables excluded from multivariate analyses as uninformative, and abbreviations given in Supplemental Data S3.

Measurements of 71 cranial and dental variables, for 90 extant adult emballonurid specimens, were collected for quantitative analysis (Fig. 1; Supplemental Data S3 (Fig. S3.1; Tables S3.2 and S3.3)). Skull characters were those used by Chimimba & Kitchener (1991), and tooth and dentary characters followed Hand (1985). Mean measurements of left and right dentitions were used. Because the raw data from Chimimba & Kitchener’s (1991) morphometric analysis of Australian emballonurids was not published, and has subsequently been lost, measurements of many of the same specimens used in that study were repeated but with additional dental characters included.

Specimens were measured at the University of New South Wales using a Wild 5MA stereomicroscope with Wild MMS235 Digital length measuring set (accurate to 0.01 mm) and Mitutoyo M.N. 85 dial calipers (accurate to 0.05 mm) for larger measurements (greatest skull length, basicranial length and cranial height). Specimens with prefix AM M were measured at the Australian Museum using a Leica MZ95 stereomicroscope with graticule (accurate to 0.01 mm) and digital TESA CAL IP65 calipers (accurate to 0.01 mm). Measurements produced by these devices were cross-checked for repeatability by measuring a subset of emballonurid specimens at both institutions. Fossil specimens were imaged using a QUANTA 200 Scanning Electron Microscope in the Mark Wainwright Analytical Centre, UNSW Sydney, and modern specimens by Leica M205 C stereo microscope with Leica DFC290 digital microscope camera or Nikon DSLR D5100 digital camera.

### Analytical methods

Univariate analyses of modern and fossil species were conducted to examine levels of variability in craniodental measurements within modern taxa and the fossil sample.

The Coefficient of Variation (CV) was used to compare variability in the fossil sample with that found in modern emballonurid species: $CV = \textstyle{{100s} \over {\rm X}}$ (*x* is the mean and *s* is the standard deviation).

The statistical program PAlaeontological STatistics (PAST; Davis, 1986; Hammer, Harper & Ryan, 2001) was used for multivariate analysis. Several variables from Chimimba & Kitchener (1991) and Hand (1985) were excluded from final analyses due to the high degree of missing data in fossil or modern samples, or because they were identified as uninformative in preliminary analyses (Supplemental Data S3 (Fig. S3.1; Glossary)).

To test for sexual dimorphism in craniodental measurements of Australian emballonurid taxa, a PERMANOVA (non-parametric MANOVA or NPMANOVA) of the best-represented species in the study, *T. georgianus*, was performed on 43 characters (Table 1). Due to the increased likelihood of misidentification in specimens both from the east of Mount Isa and from its surrounding area, this analysis was restricted to 32 specimens from west of Mount Isa (2 of 34 available specimens were not used due to missing sex data; Supplemental Data S1 (Table S1.1)). PERMANOVA was considered the best analysis to test for sexual dimorphism, instead of parametric ANOVA or MANOVA, due to: (1) 35% of characters being non-normally distributed in raw format and 40% when log-transformed; (2) the data failing Box’s *M* test for homoscedasticity (raw data: *F* = −7.18, *p* = 0; log-transformed: *F* = −1.56, *p* = 0); and (3) the data failing multivariate normality tests determined on PCA scores (raw data: Mardia kurtosis = −7.57, *p* <<< 0.001, Doornik & Hansen omnibus test, Ep = 105.7, *p* = 0.0005; log transformed data: Mardia kurtosis = −7.57, *p* <<< 0.0001, Doornik & Hansen omnibus test, Ep = 91.11, *p* = 0.009). Homogeneity of variances, an assumption of PERMANOVA, was confirmed using the ‘permutest.betadisper’ function (a multivariate analogue of Levene’s test) in R (*F* = 1.60, *p* = 0.24; Anderson (2006); R Core Team (2017)).

**Table 1:** PERMANOVA of *Taphozous georgianus* (from west of Mount Isa only) to test for sexual dimorphism. Analysis of 43 craniodental characters across 11 variables.

Variables	Number of specimens (M/F)	*F*-value	*p*
All cranial/dental (43 chars)	14/18	0.65	0.63
C^1^ L, W	14/17	0.17	0.85
P^4^ L, W	14/18	0.03	0.97
M^1^ L, W	14/18	1.50	0.23
M^2^ L, W	14/18	0.19	0.80
M^3^ L, W	14/18	2.17	0.15
C_1_ L, W	14/16	0.79	0.45
P_4_ L, W	14/18	0.90	0.43
M_1_ L, W	14/17	0.80	0.42
M_2_ L, W	14/18	0.15	0.15
M_3_ L, W	14/18	0.29	0.29

A Principal Components Analysis (PCA) was undertaken to investigate whether currently recognised modern Australian emballonurid species could be differentiated in morphospace, using craniodental characters, and to investigate multivariate differences within fossil taxa suggested by univariate analyses as well as qualitative analyses (see “Systematics”). All characters for all modern taxa were included in these analyses. Further analyses, combining modern and fossil taxa, included amalgamated cranial and dental characters, as well as a dataset of dental characters alone (canines, premolars and molars).

Distributions of *T. georgianus* and *T. troughtoni* overlap in north-western Queensland, with both species occurring in the Mount Isa Inlier bioregion (Environment Australia, 2000). The identities of 18 *Taphozous* specimens (identified as *T. georgianus*) collected previously from this geographic area, and currently held in museum collections, were checked in this study using Canonical Variate Analysis (CVA) classification with a training set of *T. troughtoni*, *T. georgianus* (only specimens from west of Mount Isa), *T. australis* and *T. hilli* (Table 2). The training data is used to determine discriminant functions that exhibit maximum variation between a priori groups, while also minimising variation within these groups, which then allow for classification of unknown specimens (Harper & Owen, 1999). Two additional specimens (AM M27892 and AM M8550), previously identified in the PCA as outliers, were also included.

**Table 2:** Loadings for CVA of *Taphozous* spp. Axis 1 and Axis 2 account for 69.9% and 16.6% of variation respectively.

Character	Axis 1	Character	Axis 2
Dentary length (DL)	0.14	Distance outside promontorium (OP)	−0.12
Basicranial length (BL)	0.13	Braincase width (BW)	−0.09
Greatest skull length (GL)	0.13	Least interorbital width (LOW)	−0.08
Rostrum length (ROL)	0.1	Greatest skull length (GL)	−0.07
Palatal length (PL)	0.1	Mastoid width (MW)	−0.07

Canonical Variate Analysis (CVA) was used on the complete character dataset for modern Australian emballonurids to investigate generic and species boundaries, particularly in the *T. georgianus*/*T. troughtoni* complex, and on dental variables alone for fossil and modern species combined. CVA allowed for examination of morphometric differentiation of taxa, determination of potentially useful discriminating variables, and classification of fossil specimens.

## Systematics

Class Mammalia Linnaeus, 1758

Order Chiroptera Blumenbach, 1779

Family Emballonuridae Gervais, 1855

Subfamily Taphozoinae Jerdon, 1867

Genus *Taphozous* E. Geoffroy, 1818

*Taphozous georgianus* Thomas, 1915

**Figure 2:**
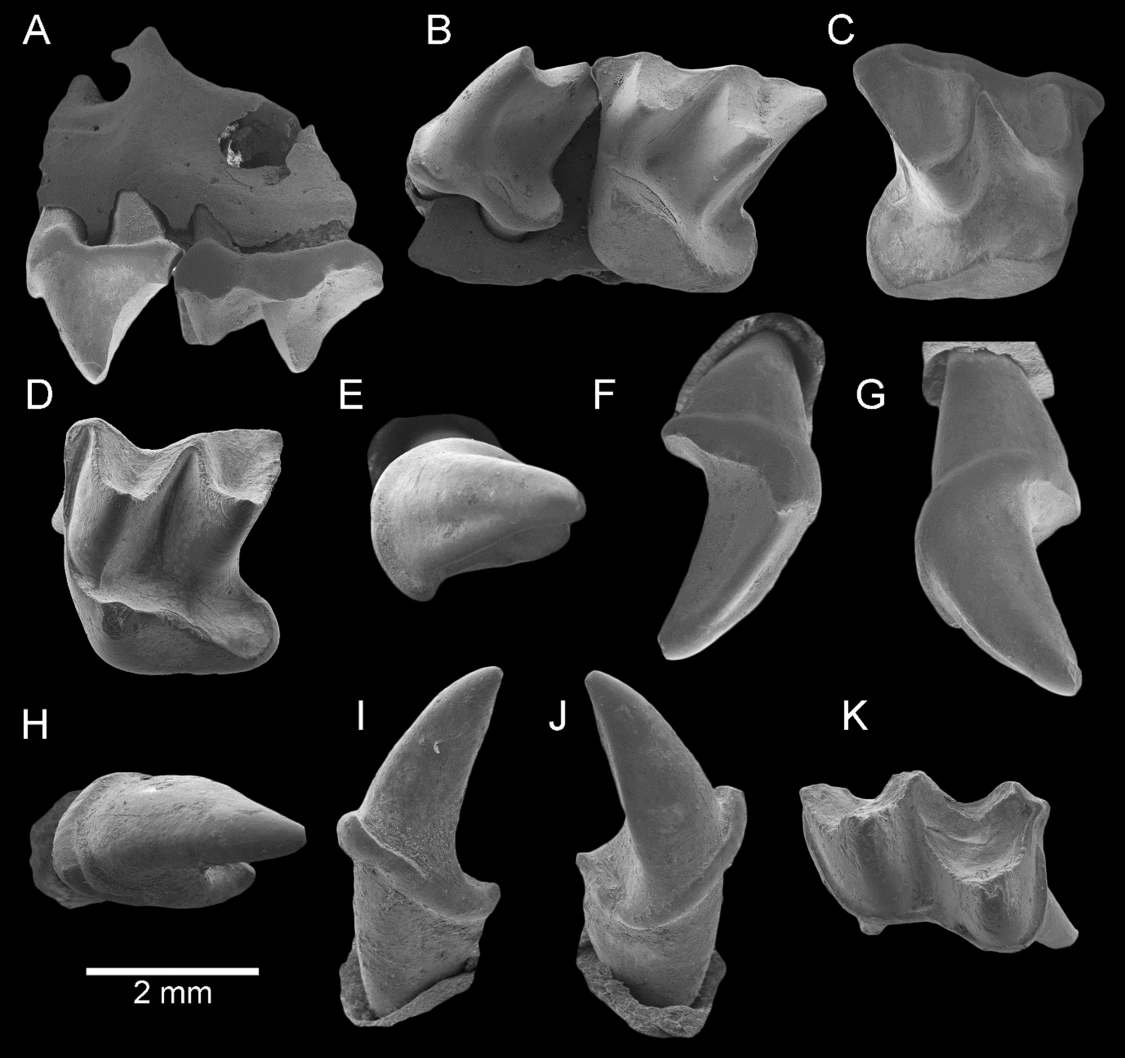
*Taphozous georgianus*. Fossil material from Rackham’s Roost Site, Riversleigh WHA, north-western Queensland. AR21229, left maxillary fragment with P^4^–M^1^ and infraorbital foramen: (A) buccal view; (B) oblique-lingual view. AR21209, right M^1^: (C) occlusal view. AR21230, left M^2^: (D) oblique-lingual view. AR21240, left C^1^: (E) occlusal view; (F) oblique-lingual view; (G) oblique-buccal view. AR21225, right C_1_: (H) occlusal view; (I) lingual view; (J) buccal view. AR21220, left M_1_: (K) oblique-occlusal view. Scale = 2 mm.

**Figure 3:**
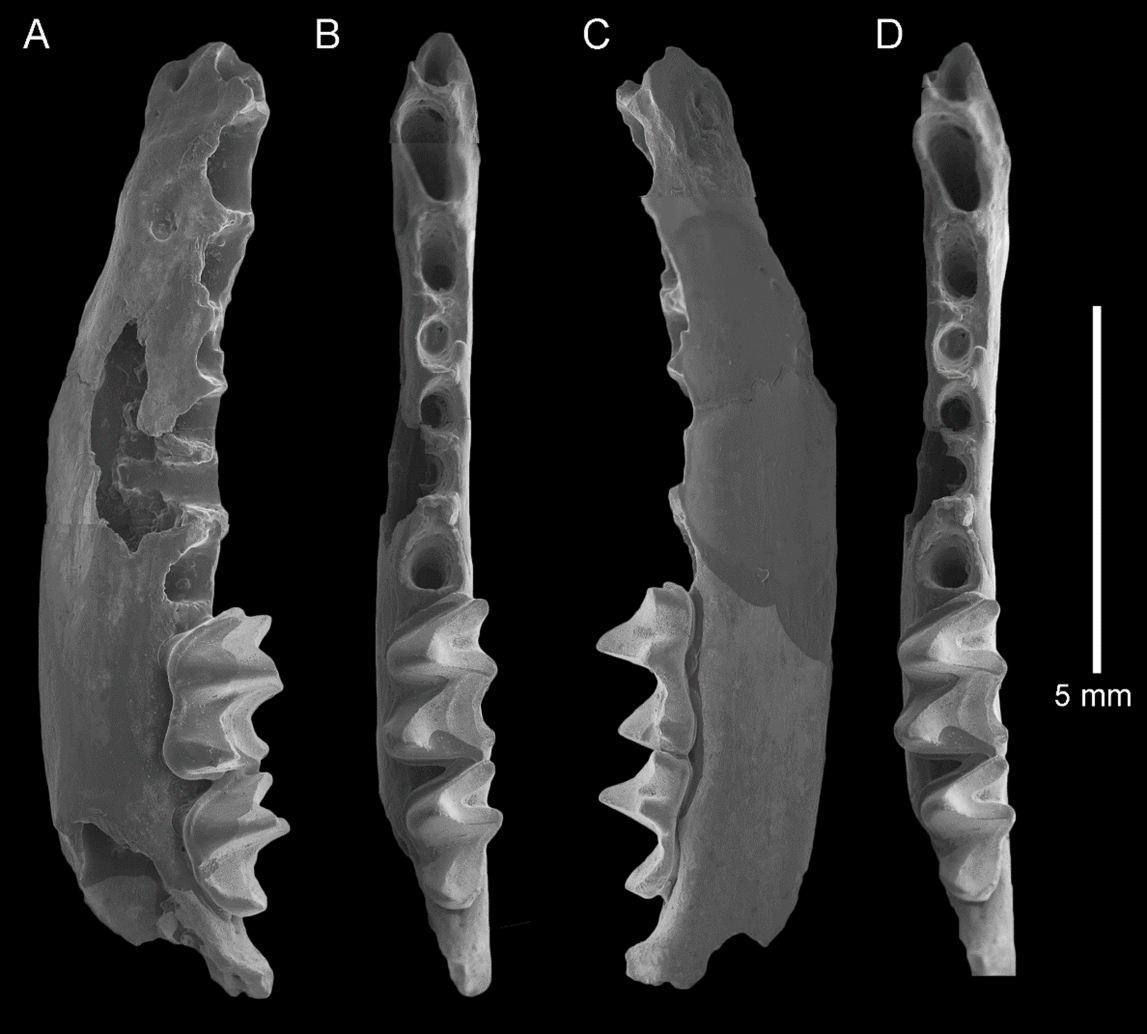
*Taphozous georgianus*. Fossil material from Rackham’s Roost Site, Riversleigh WHA, north-western Queensland. AR21243, left dentary fragment with M_2-3_ and alveoli for I_1-2_, C_1_, P_3-4_, M_1_: (A) buccal view; (B) and (D) stereopairs, occlusal view; (C) lingual view. Scale = 5 mm.

**Figure 4:**
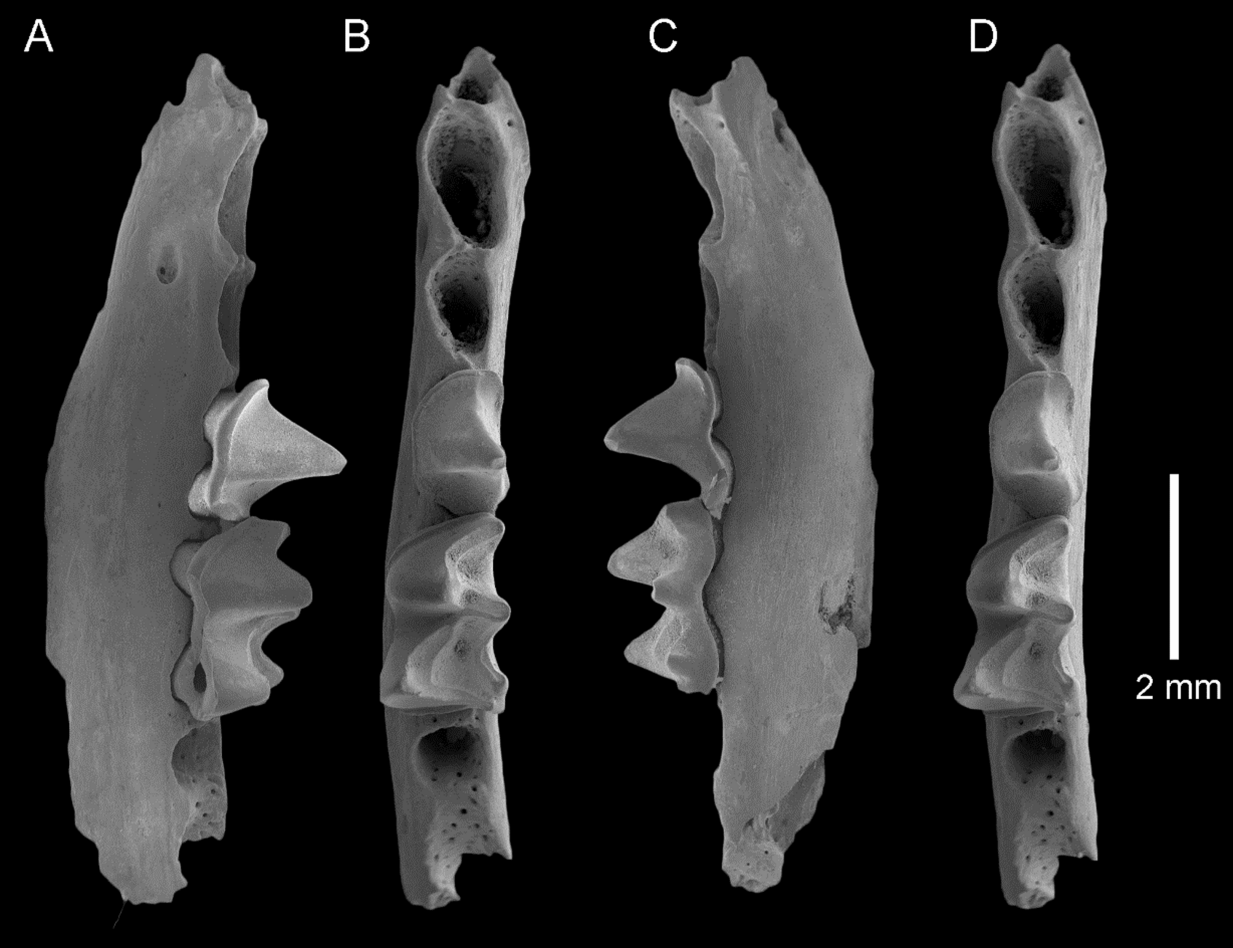
*Taphozous georgianus*. Fossil material from Rackham’s Roost Site, Riversleigh WHA, north-western Queensland. QM F23866, left dentary fragment with P_4_ and M_1_ and alveoli for I_1-2_, C_1,_ P_3_ and M_2_: (A) buccal view; (B) and (D) stereopairs, occlusal view; (C) lingual view. Scale = 2 mm.

**Figure 5:**
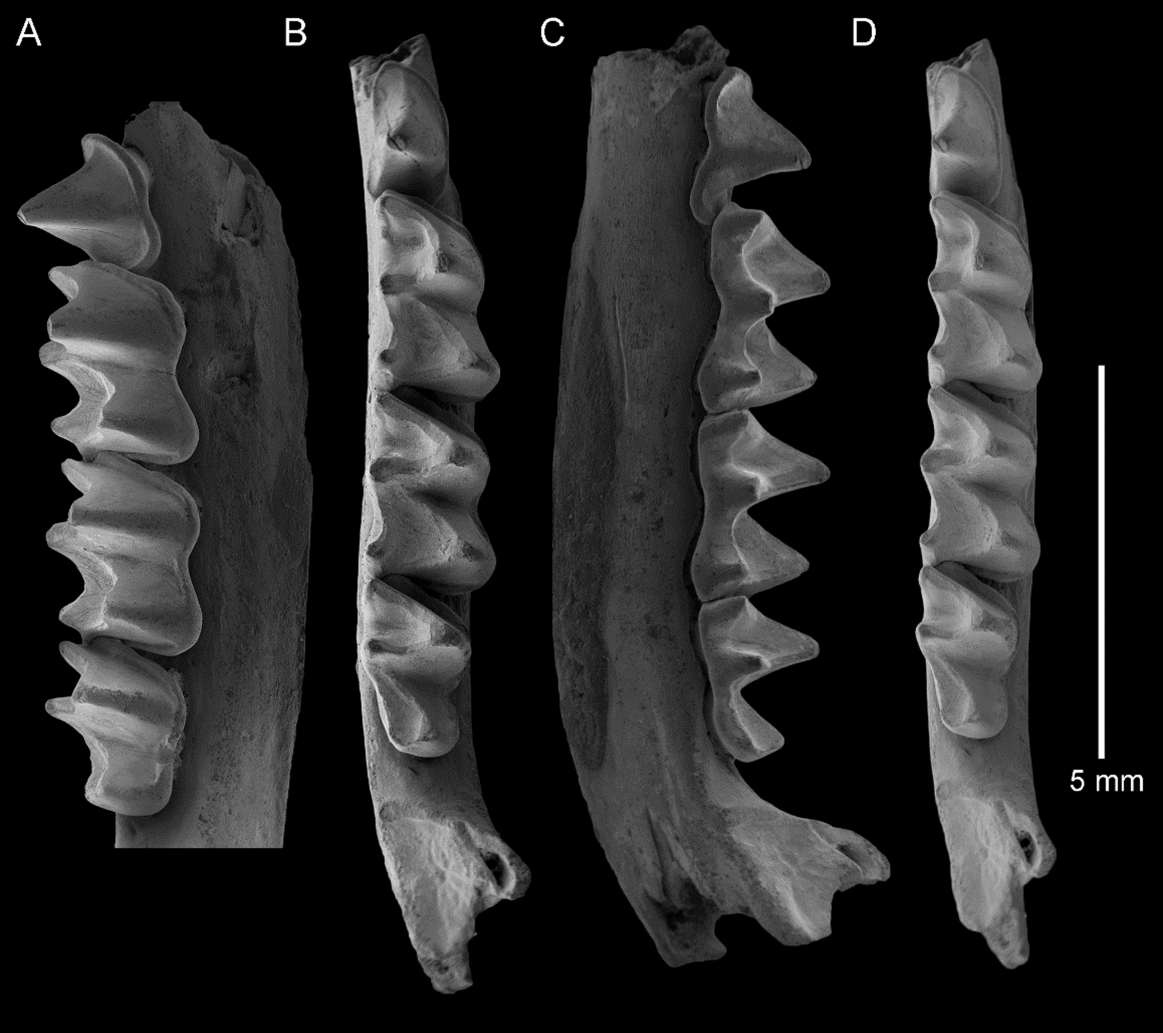
*Taphozous georgianus*. Fossil material from Rackham’s Roost Site, Riversleigh WHA, north-western Queensland. QM F23628, right dentary fragment with P_4_–M_3_: (A) buccal view; (B) and (D) stereopairs, occlusal view; (C) lingual view. Scale = 5 mm.

Figures 2–5; Supplemental Data S2 (Fig. S2.1)

**Referred material**. AR21191, left C^1^; AR21192, left M_3_; AR21193, left M_2_; AR21194, right M^2^; AR21195, left M^1^; AR21196, left M^2^; AR21197, left M_2_; AR21198, right M^2^; AR21199, right M^2^; AR21200, left M_2_; AR21201, right M^2^; AR21202, right C^1^; AR21203, left C_1_; AR21204, right C^1^; AR21205, right C^1^; AR21206, left M^2^; AR21207, left C^1^; AR21208, right M_2_; AR21209, right M^1^ (Fig. 2C); AR21210, right M_2_; AR21211, right C_1_; AR21213, right C^1^; AR21215, left C^1^; AR21216, left C_1_; AR21218, left C^1^; AR21219, right P_4_; AR21220, left M_1_ (Fig. 2K); AR21221, left M^2^; AR21222, right C^1^; AR21223, left M^2^; AR21224, right M^2^; AR21225, right C_1_ (Figs. 2H–2J); AR21226, left M_3_; AR21227, right C_1_; AR21229, left maxillary fragment with P^4^–M^1^ and infraorbital foramen (Figs. 2A and 2B); AR21230, left M^2^ (Fig. 2D); AR21231, right M^1^; AR21232, right dentary fragment with M_3_ and partial alveolus from M_2_; AR21234, left M^2^; AR21235, left M^1^; AR21236, left M^2^; AR21237, left M_3_; AR21238, left M^2^; AR21239, right C^1^; AR21240, left C^1^ (Figs. 2E–2G); AR21242, right M^1^; AR21243, left dentary fragment with M_2-3_ and alveoli for I_1-2_, C_1_, P_3-4_, M_1_ (Figs. 3A–3D); QM F23628, right dentary fragment with P_4_–M_3_ (Figs. 5A–5D); QM F23840, left M^1^; QM F23843, left C^1^; QM F23844, right M^1^; QM F23847, left M_2_; QM F23848, right M^1^; QM F23849, right M^2^; QM F23853, left dentary fragment with M_2-3_ and partial alveolus for M_1_; QM F23854, left M^1^; QM F23857, left M_3_; QM F23865, left M^1^; QM F23866, left dentary fragment with P_4_ and M_1_ and alveoli for I_1-2_, C_1_ and P_3_ (Figs. 4A–4D); QM F23867, left dentary fragment with M_2-3_.

**Fossil locality**. Rackham’s Roost Site, Riversleigh World Heritage Area, north-western Queensland. Precise coordinates are available on application to the Queensland Museum, Brisbane. Rackham’s Roost Site is early Pleistocene in age based on radiometric dating of associated speleothem (~2.1 Ma; Woodhead et al., 2016).

**Species attribution**. Differs from *T. troughtoni*, *T. australis*, *T. kapalgensis* and *T. hilli* (Supplemental Data S2) in the following combination of features: only shallow concavity in face between C^1^ and P^4^; infraorbital foramen low on face, approximately one premolar (P^4^) height dorsal to toothrow; C^1^ short and narrow; P^3^ evidently as great as one-third P^4^ crown area; P^4^ long but narrow; dentary relatively shallow, its ventral margin without accentuated chin process beneath P_4_; M_3_ with talonid shorter than trigonid.

With extant *T. georgianus*, it shares C_1_, C^1^, M^1^, M_2_ and M^2^ that are shorter relative to those in *T. troughtoni*, but longer than in *T. hilli* and a wider C^1^, M_1_ and M_2_, as well as a narrower M^1^ and M^2^, relative to *T. australis*.

**Remarks**. These specimens are referred to *Taphozous* rather than *Saccolaimus* because the ventral margin of the dentary beneath the premolars is slightly concave rather than convex (but see *S. flaviventris*; Supplemental Data S2 (Fig. S2.4)), it has a single (rather than double) infraorbital foramen, the orbital rim is rounded rather than conspicuously sharp, alveolus size indicates P^3^ is relatively small (less than one-third posterior premolar crown area), P^4^ is t-shaped rather than rectangular, M^1^ has a well-developed rather than reduced parastylar area, and M_3_ is relatively short.

**Description**. Maxillary fragment AR21229 preserves part of the anterolateral face of the skull, including the infraorbital foramen. The single infraorbital foramen is relatively large and developed dorsal to P^4^. The rounded ventral rim of the orbit extends from the posterior half of P^4^, above its dorsal margin, to at least the posterior face of M^1^. A depressed fracture, dorsal to M^1^ (Fig. 2A), suggests damage caused by the tooth of a predator (see “Discussion”).

C^1^ is elliptical to semicircular in basal outline with lingual margin flatter. It has one principal cusp, recurved posteriorly, and an elongate posterior heel comprising nearly half the crown length. The anterobuccal face of the paracone is convex and the posterolingual face flat to convex. A longitudinal groove runs down the anterior face of the paracone. A cingulum is continuous anteriorly, lingually and posteriorly (surrounding the heel) and attenuates buccally. The lingual cingulum is taller anteriorly than posteriorly. A well-developed anterolingual cusp is always present and two much smaller posterior cingular cuspules are usually present. Typically, a further two cusps are developed in the lingual cingulum (e.g. AR21207); a third lingual cingular cusp is sometimes present (e.g. AR21205).

P^4^ is boomerang or T-shaped in basal outline. It has a single principal cusp nearly as tall as the metacone on M^1^, with convex anterior and buccal faces and a concave lingual face, with a blade running posterobuccally from the apex. It is nearly as wide as the anterior face of M^1^ (e.g. AR21229). The tooth has a posterolingual heel in which a basin is well developed. A cingulum is continuous anteriorly, lingually and posteriorly but dissipates buccally towards the crown’s broadest convexity which occurs at about mid-length. A small but distinct anterior cingular cusp is present. The tooth has three roots, one anterior, one on the buccal margin and the third posterolingual (with respect to the toothrow).

M^1^ is dilambdodont. It is trapezoidal in basal outline with posterior width greater than anterior width. The metacone is taller than the paracone, which is also well developed. The protocone is lower than the paracone and is associated with a very large posteriorly directed heel with deep basin. The protofossa basin is open posteriorly. Increasing in length are the preparacrista, postparacrista, premetacrista and postmetacrista; the last is conspicuously elongated. There is no paracingulum, but a parastylar shelf is well developed. A metastylar shelf is evident in some unworn specimens (e.g. AR21235). A pre-ectoflexus is developed between the parastyle and mesostyle, and a very shallow post-ectoflexus between mesostyle and metastyle. A narrow metacingulum is present. The preprotocrista extends anterobuccally to reach the base of the anterior flank of the paracone. The postprotocrista is continuous with the cingulum surrounding the heel. In some unworn specimens (e.g. AR21235), an additional short lingual cingulum is developed near the base of the crown between the protocone and lingual swelling of the heel. The posterior margin of the heel is rather square.

M^2^ is described in so far as it differs from M^1^. M^2^ is squarer in basal outline, with posterior width only slightly greater than anterior width. The paracone is taller and better developed but is still shorter than the metacone. A paracingulum is better developed and metacingulum is less well developed or absent (e.g. AR21230). The pre-ectoflexus is deeper but the post-ectoflexus absent.

M^3^ is unknown.

The dentary is represented by several specimens including those shown in Figs. 3–5. The dentary is relatively shallow, being approximately one molar height deep. Its ventral margin lacks the accentuated chin process beneath P_4_ that is commonly found in modern *Taphozous* species (Supplemental Data S2), as well as the associated sharp change in depth between anterior and posterior portions of the dentary. A mental foramen occurs between and ventral to the alveoli for C_1_ and P_3_. Smaller foramina occur further anteriorly, the largest of these occurring ventral to the alveolus for I_1_. The dentary symphysis extends posteriorly to the posterior margin of C_1_. The posterior dentary is best represented by QM F23628, but even in this specimen most of the ascending ramus, masseteric fossa, angular process and condyle are not preserved (Fig. 5).

Alveoli are preserved for the anterior dentition in AR21243 (Fig. 3) and QM F23866 (Fig. 4). As in all other Australian emballonurids, two incisors were present, a relatively short and narrow C_1_ and relatively large, single-rooted P_3_ almost as large as the posterior premolar P_4_. Of these, only the C_1_ is represented by teeth in the fossil sample.

C_1_ is elliptical to semicircular in basal outline with lingual margin relatively flat. Its tall central cusp is only slightly recurved posteriorly, with convex anterior face and flattened posterior face meeting at posterolingual and posterobuccal crests running from apex to base. In occlusal and buccal views, a conspicuous heel represents approximately one third overall tooth length. A thin cingulum surrounds the crown but attenuates buccally. A small cingular cusp occurs anteriorly and typically two posteriorly.

P_4_ is two-rooted, semi-circular to rectangular in basal outline with lingual margin flatter than buccal margin, and anterior width slightly greater than posterior width. It has a single central cusp that is taller than any molar cusp. Crests run from the apex anteriorly and to the posterolingual corner of the tooth, the latter crest being more blade-like than the former. A cingulum surrounding the tooth is more or less continuous with no obvious cingular cusps.

M_1_ is rectangular in basal outline and has two roots. The trigonid is longer than the talonid and its width is approximately two thirds of talonid width. The trigonid opens lingually, with the pre- and postprotocristid forming nearly a right angle. The paracristid is conspicuously longer than the metacristid. The talonid is walled lingually by a tall entocristid and well developed postmetacristid and pre-entocristid. The tooth displays the nyctalodont pattern in which the hypocristid (=posthypocristid) runs from the hypoconid to the hypoconulid, bypassing the entoconid. In unworn specimens, this pattern and the separation of entoconid and hypoconulid is clear (AR21220; Fig. 2K), but in worn specimens the pattern appears to be almost submyotodont (e.g. QM F23628; Fig. 5). The paraconid, metaconid and protoconid are approximately equally tall, and the entoconid just shorter; all are conspicuously taller than the hypoconid and small hypoconulid. The cristid obliqua joins the metaconid lingual of centre and ventral to the point at which the premetacristid and postmetacristid meet. Anterior and posterior cingulids are present, with a narrow buccal cingulum dissipating around the base of the protoconid and hypoconid.

M_2_ is described in so far as it differs from M_1_. The trigonid is more anteroposteriorly compressed and not as widely open lingually, with the angle made between pre- and postprotocristid being closer to 60 degrees. The paracristid and metacristid are more equal in length. The trigonid is shorter than the talonid and is approximately three quarters talonid width. The buccal cingulid is absent at the base of the hypoconid. The cristid obliqua meets the metaconid further lingually (more lingual than directly ventral to the junction of the components of the metacristid).

M_3_ is described in so far as it differs from M_1-2_. M_3_ is reduced in length, width and height with respect to M_1-2_. The trigonid is shorter, narrower and more anteroposteriorly compressed, and the talonid and its cusps are much reduced. The protoconid is approximately twice the height of the hypoconid, and there is no distinct entoconid or hypoconulid. The cristid obliqua meets the metaconid further lingually, and the talonid basin is oval or tear-shaped rather than triangular.

Genus *Taphozous* E. Geoffroy, 1818

*Taphozous troughtoni* Tate, 1952

**Figure 6:**
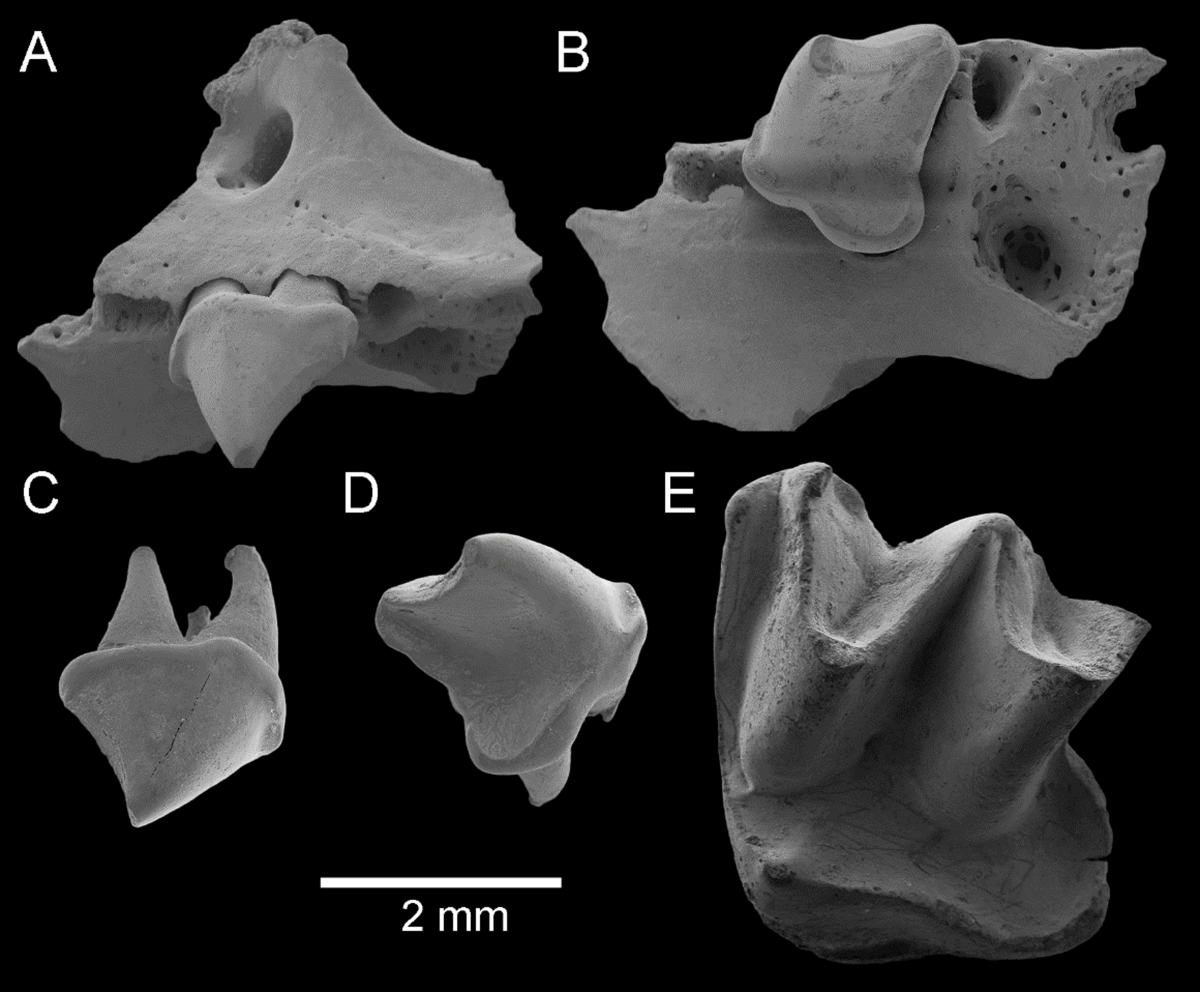
Fossil material from Rackham’s Roost Site, Riversleigh WHA, north-western Queensland. *Taphozous troughtoni*. QM F23868, left maxillary fragment with P^4^ and alveoli for P^3^, M^1^ and partial alveolus for C^1^: (A) oblique-buccal view; (B) oblique-lingual view. QM F23845, right P^4^: (C) oblique-buccal view; (D) oblique**-**lingual view. *Saccolaimus* sp. QM F23846, left M^2^: (E) oblique-occlusal view. Scale = 2 mm.

Figures 6A–6D; (Supplemental Data S2 (Fig. S2.2))

**Material**. QM F23868, left maxillary fragment with P^4^ and alveolus for P^3^ and partial alveolus for C^1^ (Figs. 6A and 6B); QM F23845, a right P^4^ (Figs. 6C and 6D).

**Locality**. Rackham’s Roost Site, Riversleigh World Heritage Area, north-western Queensland. The precise coordinates of the type locality are available on application to the Queensland Museum, Brisbane.

**Age and distribution**. Rackham’s Roost Site is early Pleistocene in age based on radiometric dating of associated speleothem (~2.1 Ma; Woodhead et al., 2016).

**Description**. The alveolus for C^1^, which is a relatively narrow tooth, would have been orientated roughly parallel to the cheek tooth row, rather than medially. An indentation in the face between C^1^ and P^4^, which is typically found in emballonurids (Supplemental Data S2), appears to have been shallow. A single, large infraorbital foramen is developed dorsal to P^4^. P^3^ is not represented in the fossil sample but appears to have been a relatively small, single-cusped tooth, evidently less than one-third of P^4^ crown area. P^4^ is similar in overall morphology to that tooth in the Rackham’s Roost population of *T. georgianus* (e.g. AR21229) but can be distinguished by its smaller width and greater length, although being approximately as wide as in modern *T. georgianus*. These P^4^ specimens also fall within the width range observed in modern *T. troughtoni*, although they are slightly longer (Supplemental Data S3).

Genus *Saccolaimus* Temminck, 1838

*Saccolaimus* sp.

Figure 6E

**Material**. QM F23846, a left M^2^ (Fig. 6E)

**Locality**. Rackham’s Roost Site, Riversleigh World Heritage Area, north-western Queensland. The precise coordinates of the type locality are available on application to the Queensland Museum, Brisbane.

**Age and distribution**. Rackham’s Roost Site is early Pleistocene in age based on radiometric dating of associated speleothem (Woodhead et al., 2016).

**Remarks**. *Saccolaimus* sp. differs from *S. saccolaimus* and *S. mixtus* in its much larger molar size (e.g. M^2^L and M^2^W, Supplemental Data S3 (Tables S3.1 and S3.2) and Supplemental Data S4)). It differs from *S. flaviventris* in M^2^ being slightly longer (Supplemental Data S2 (Fig. S2.4) and Supplemental Data S3 (Table S3.2)), and in also having a paracingulum, a paraloph, and a depression (with cingulum) between the protocone and heel and better developed parastyle.

**Description**. M^2^ is dilambdodont and would have had three roots; the posterobuccal root and metastylar region of the crown are missing. The tooth is otherwise intact and relatively unworn. It appears to have been approximately square in basal outline. The metacone is just taller than the paracone. The protocone is lower than both, and protofossa basin opens posteriorly. A paracingulum, continuous from parastyle to preprotocrista, is clearly present. The postprotocrista is continuous with a cingulum surrounding a broad, posteriorly directed heel with deep basin, which in turn is continuous with a narrow metacingulum. A short lingual cingulum is developed near the base of the crown between the protocone and the heel. A buccal shelf (including cuspidate or crestiform mesostyle) is evidently absent. A deep, v-shaped pre-ectoflexus occurs between the parastyle and mesostyle; because of damage it is not clear whether a post-ectoflexus was developed. A short, curved paraloph extends from the base of the paracone to the paracingulum.

## Results

### Quantitative analysis of modern Australian emballonurids

**Figure 7:**
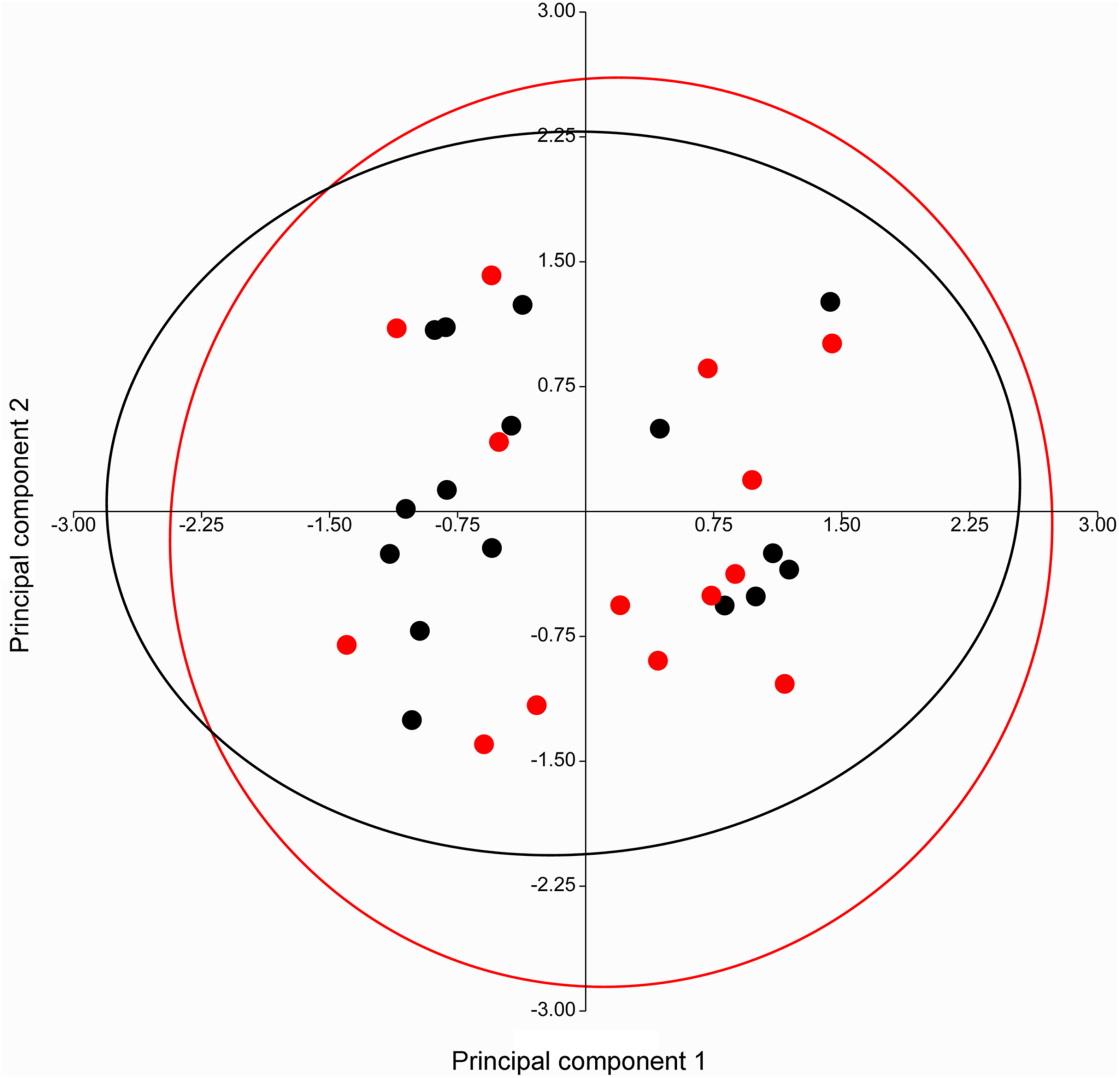
PCA of craniodental characters for *T. georgianus* specimens from west of Mount Isa. Highly overlapping ellipses support lack of sexual dimorphism as indicated by PERMANOVA (Table 1). Red—males; Black—females. Ellipses are 95% confidence limits. Principal components 1 and 2 represent 27.0% and 23.1% of variation, respectively.

A PERMANOVA (using the Euclidean similarity index) performed on specimens of *Taphozous georgianus*, from the west of Mount Isa, revealed no sexual dimorphism in 11 analyses incorporating from 2 to 43 cranial and dental characters (Table 1), justifying combining males and females in subsequent analyses. The pattern of clustering in a PCA of craniodental characters supported these results, with substantial overlapping of 95% elliptical confidence regions and centroids (Fig. 7).

Simpson, Roe & Lewontin (1960) proposed that, in individual linear measurements of anatomical elements of mammals, CV values between 4 and 10 indicate reasonably unified samples (i.e. single species), while values lower than 4 suggest sample size was inadequate to assess variability and values greater than 10 indicate that the sample probably represents more than one species (see “Discussion” in Cope & Lacy, 1992, 1995; Plavcan & Cope, 2001). Minimal variability was found within each modern emballonurid species across 71 measurements of the Coefficient of Variation (CV) (Supplemental Data S3 (Table S3.2)). Between 78% (*S. flaviventris*) and 96% (*T. troughtoni*) of characters within each species examined, with a sufficient sample size (CV > 4), were within the range suggesting a unified sample (CV < 10). M^3^ characters (BUC L, LIN L and W), however, displayed considerable variability, with values of up to 41.41 for example in *S. flaviventris*. Similarly, variables relating to paracristid and metacristid length exhibited greater variation. Examination of *T. georgianus* specimens from west of Mount Isa only, to reduce possibility of mis-identified *T. troughtoni*, produced similar results to the sample including all *T. georgianus* specimens (Supplemental Data S3 (Table S3.2)). The values recorded here suggest characters relating to M^3^ size and cristid length are not reliable for distinguishing Australian emballonurid species.

The fossil assemblage exhibited CV values greater than 10 for several variables (e.g. M^2^ width and heel width gave results of 11.56 and 10.57, respectively) (Supplemental Data S3 (Table S3.3)). The greatest CV values, however, were for M_3_, with all five variables giving values between 11.41 and 17.48. These results suggest that more than one species may be represented in the fossil sample.

**Figure 8:**
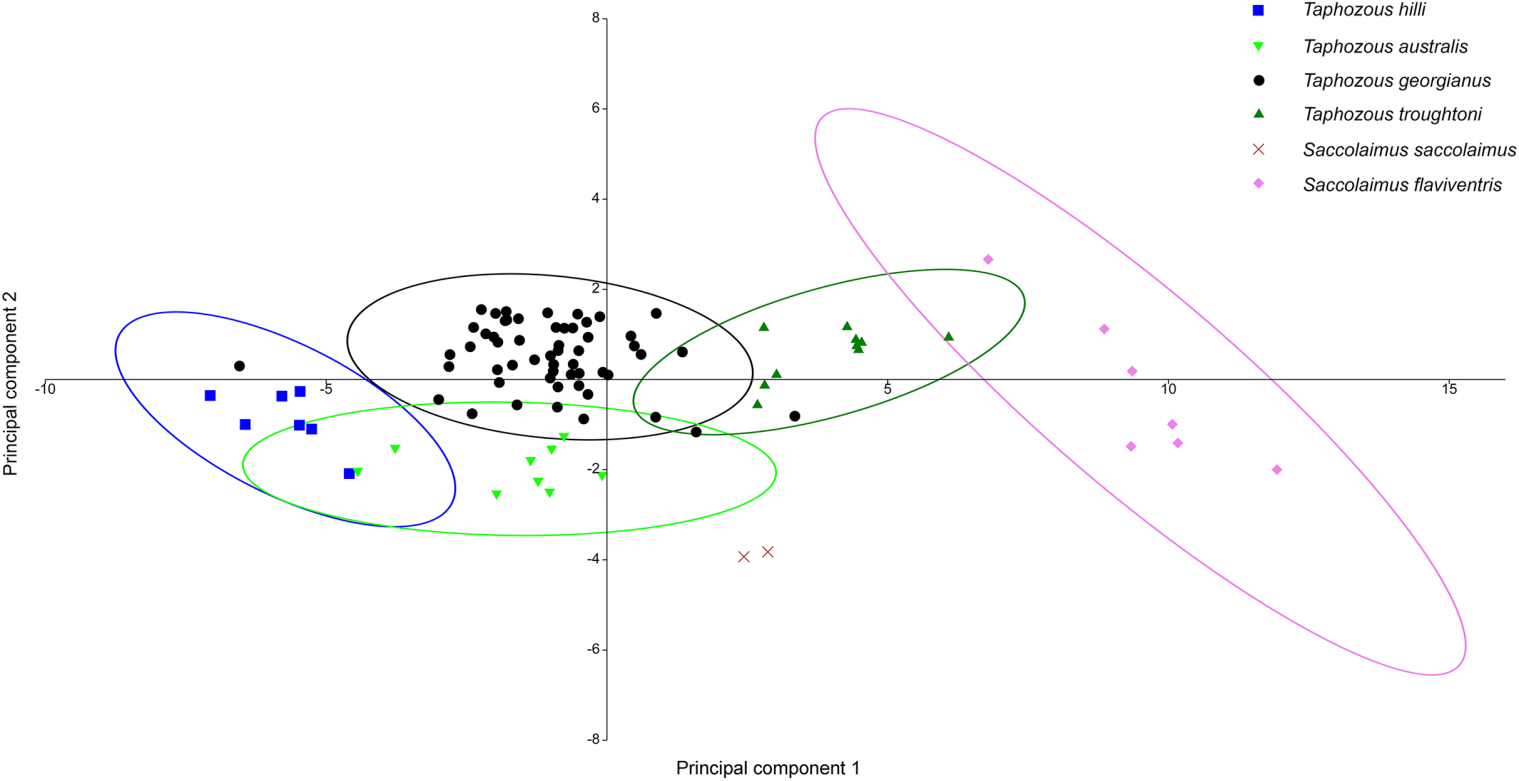
PCA of extant Australian emballonurids, using cranio-dental data. Species are mostly separated on the first two principal components. Ellipses are 95% confidence limits. Principal components 1 and 2 represent 72.1% and 8.0% of total variation, respectively.

Principal Components Analysis of modern emballonurid specimens separated most currently recognised species on the first two principal components (representing 72.1% and 8.0% of the total variation, respectively), with only partial overlap of 95% confidence ellipses among species (Fig. 8). *Taphozous hilli, T. georgianus, T. troughtoni* and *S. flaviventris* are separated primarily on PC1, with the latter only partially overlapping with *T. troughtoni. Saccolaimus saccolaimus* grouped closer to species of *Taphozous* than it did to *S. flaviventris* but, along with *T. australis*, was differentiated from other species on PC2. Characters with the highest loadings for PC1 include, in order of decreasing contribution: dentary length (DL); basicranial length (BL); greatest skull length (GL); zygomatic width (ZW); lower tooth row length (C_1_–M_3_); and rostrum length (ROL). PC2 characters with significant negative loadings are distance outside promontorium (OP), braincase width (BW) and mastoid width (MW), while characters with high positive loadings include DL, distance of M_3_ to dentary condyle (RC) and least interorbital width (LOW). No further separation was obtained using other Principal Component combinations (e.g. PC 1v3, 2v3), with even more overlap occurring between species.

**Figure 9:**
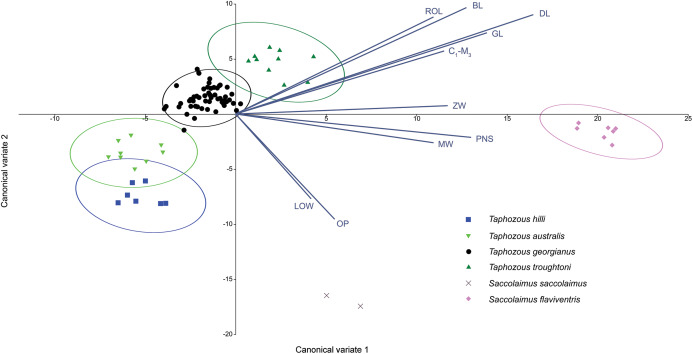
CVA of extant Australian emballonurids including biplot of the 10 variables with the highest loadings. *Taphozous* spp. mostly separated by canonical variate 2, while *Saccolaimus* spp. are separated from *Taphozous* spp. and each other by both canonical variates. Ellipses are 95% confidence limits. Canonical variate 1 represents 61.9% of variation. Canonical variate 2 represents 24.5% of variation.

A Canonical Variate Analysis (CVA) of cranial and dental variables grouped all *Taphozous* species together, while *S. saccolaimus* and *S. flaviventris* were isolated from each other, and the *Taphozous* cluster (Fig. 9). Characters with high loadings on the first axis (explaining 61.9% of the variation) are primarily cranial: DL, posterior nose-shield width (PNS), MW, GL, BL and ZW. C_1_–M_3_ is the only dental variable with a loading comparable to these. BL, DL, GL and ROL also have high loadings for axis two (24.5% of variation), while LOW and OP are inversely significant contributors to axis two loadings.

**Figure 10:**
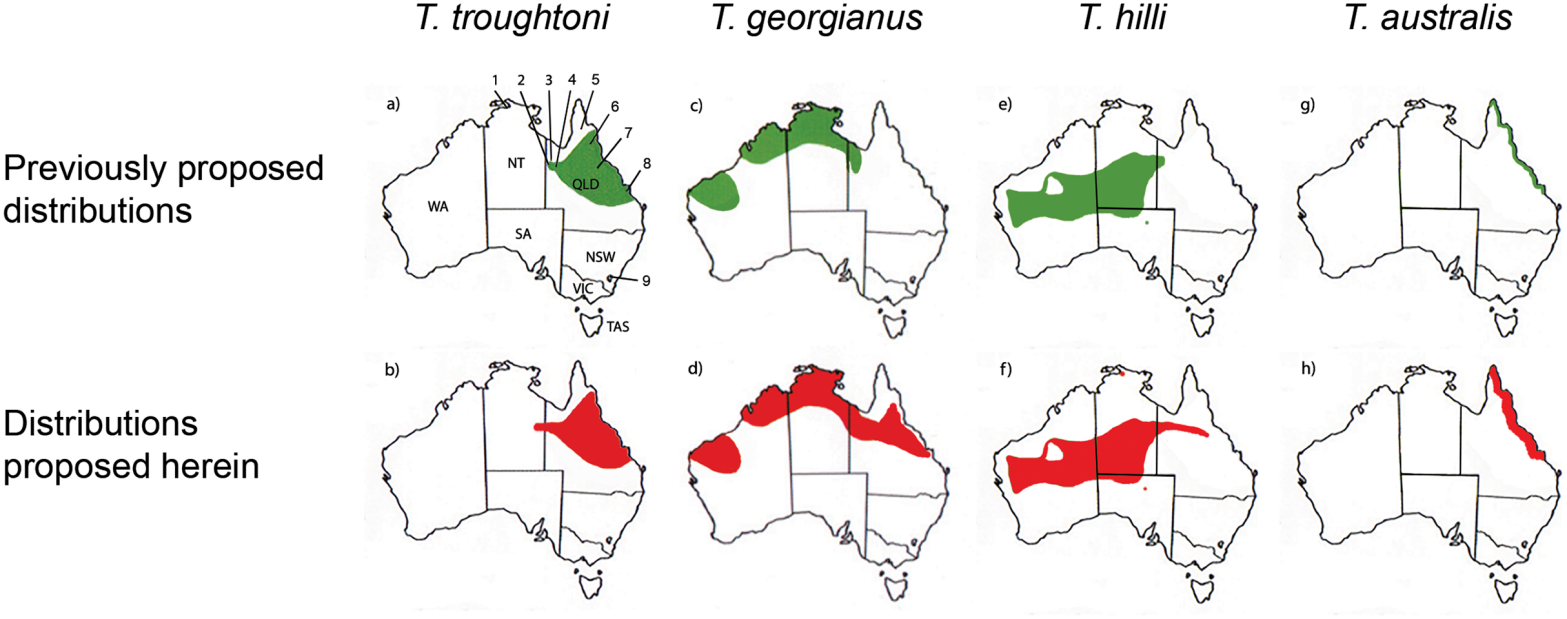
Distribution maps for *Taphozous troughtoni, T. georgianus*, *T. hilli* and *T. australis*. Previous distributions from: (A) *T. troughtoni*—Armstrong et al. (2017); (C) *T. georgianus*— Armstrong & Reardon (2017); (E) *T. hilli*—Armstrong (2020) and (G) *T. australis*—Hall, Thomson & Richards (2008). Revised distributions suggested herein: (B) *T. troughtoni*. (D) *T. georgianus*. (F) *T. hilli*. (H) *T. australis*. Geographic locations: 1—South Alligator River, NT. 2—Camooweal, QLD. 3—Mount Isa, QLD. 4—Rackham’s Roost Site, Riversleigh World Heritage Area, QLD. 5—Cape York, QLD. 6—Chillagoe, QLD. 7—Pentland, QLD. 8—Rockhampton, QLD. 9—Canberra, ACT.

**Figure 11:**
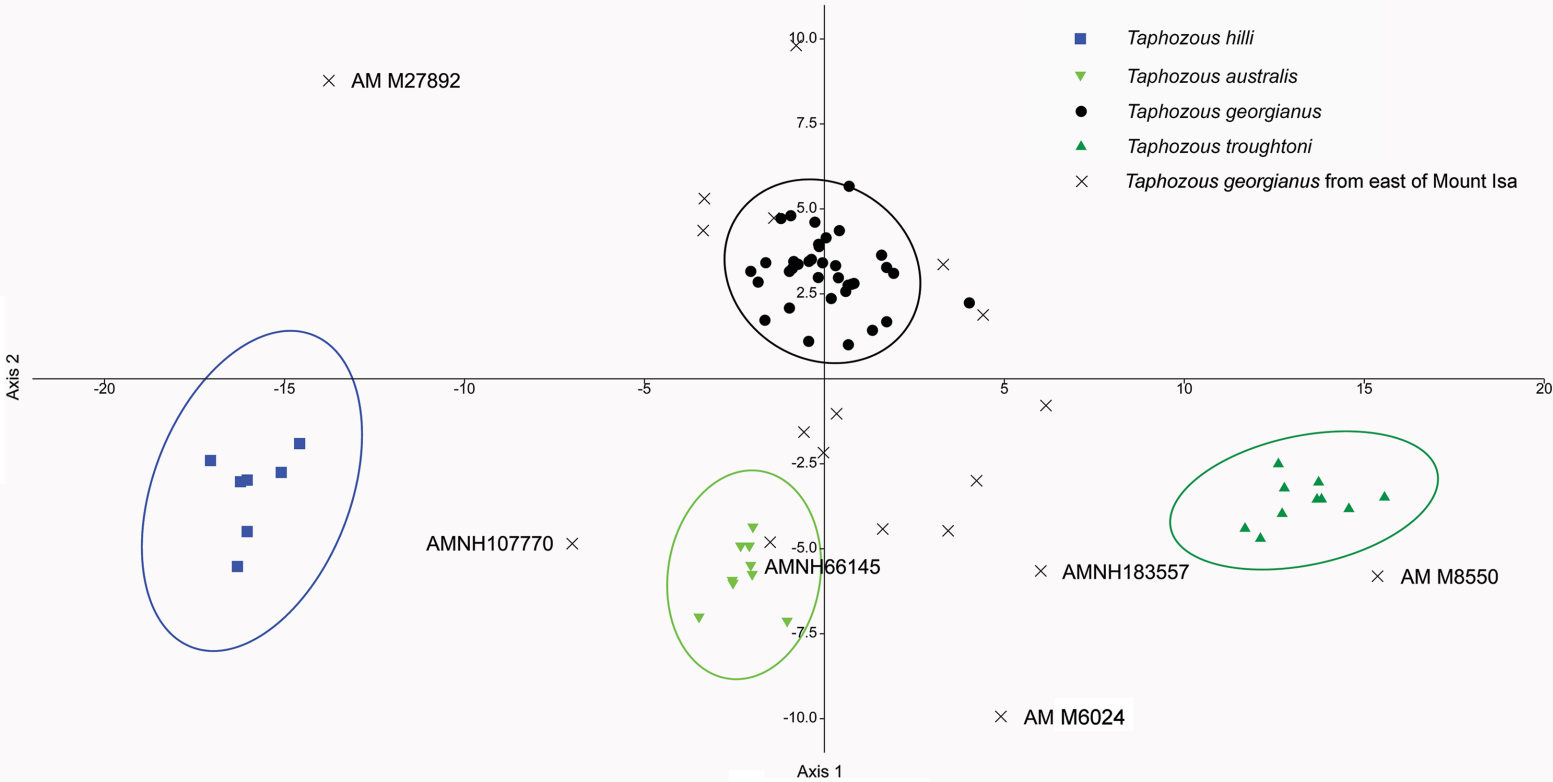
CVA for *Taphozous* spp. Specimen numbers represent specimens re-assigned to other species. 100% of *T. georgianus* from west of Mount Isa classified as *T. georgianus*. Ellipses are 95% confidence limits. Canonical variates 1 and 2 represent 69.9% and 16.6% of variation, respectively.

Reardon & Thomson (2002) significantly revised the expected distributions of *T. georgianus* and *T. troughtoni* in northern Australia. More recently, Armstrong et al. (2017) and Armstrong & Reardon (2017) re-examined the distributions for these species (Fig. 10). A CVA to confirm the identity of *T. georgianus* specimens from east of Mount Isa, using *T. troughtoni*, *T. hilli*, *T. australis* and *T. georgianus* from west of Mount Isa as training data, successfully classified 100% of specimens within the training set (Fig. 11). Of the 18 specimens from east of Mount Isa, and identified as *T. georgianus*, the CVA classified 14 as *T. georgianus*, one as *T. hilli* (AMNH107770) and three as *T. australis* (AM M6024, AMNH183557, AMNH66145). The outliers AM M8550 and AM M27892, both also originally identified as *T. georgianus*, were classified in the CVA as *T. troughtoni* and *T. hilli* respectively. AM M8550 is positioned close to the *T. troughtoni* ellipse, while AM M27892 was isolated from all other specimens, approximately equidistance from *T. hilli* and *T. georgianus* ellipses. One of the three specimens re-classified as *T. australis*, AMNH66145, plotted near the centroid of the 95% ellipse for *T. australis*, while the other two were situated between the ellipses for *T. australis* and *T. troughtoni* (Fig. 11).

The main discriminators for species of *Taphozous*, on axis one (69.9% of variation), were length-related (Table 2; Fig. 11). *Taphozous troughtoni* exhibits the longest dentary, basicranium, skull, rostrum and palate, while *T. hilli* has the shortest. Specimens of *T. georgianus* and *T. australis* were of intermediate length for these variables but were separated on axis two (16.6% of variation) by variables relating to width; *T. georgianus* has a wider basicranium and mastoid, as well as greater distance outside promontorium and interorbital width, relative to *T. australis*.

### Quantitative analysis of fossil taxa

**Figure 12:**
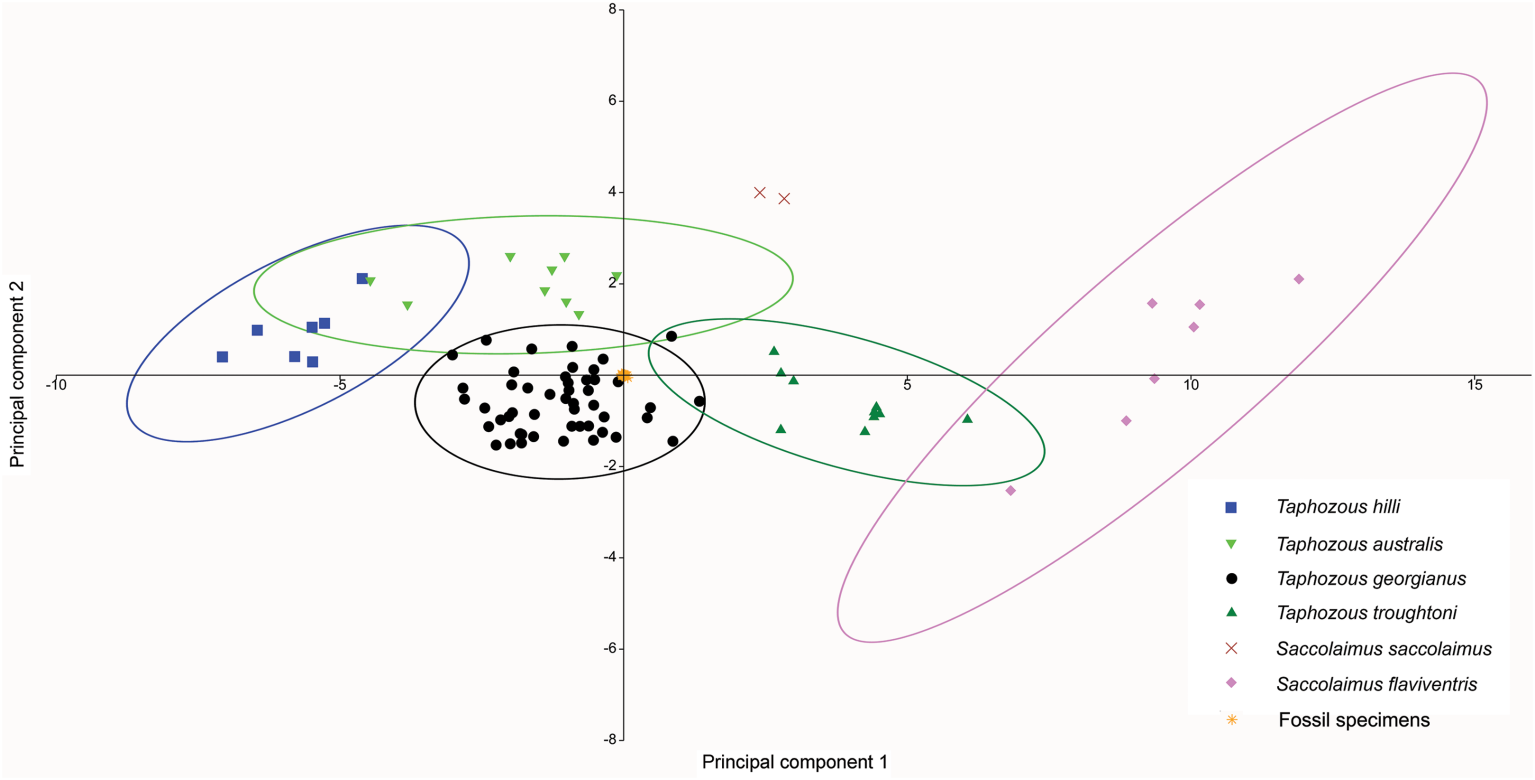
PCA of fossil and extant emballonurids, using cranial and dental data. Fossil specimens fall within *T. georgianus* ellipse but cranial data missing for most fossils. Ellipses are 95% confidence limits. PC1 and PC2 represent 71.9% and 8.2% of total variation, respectively.

At least three emballonurid taxa, *T. georgianus, T. troughtoni* and *Saccolaimus* sp., appear to be represented in the Rackham’s Roost Site fossil sample. In a PCA of extant and fossil specimens, excluding six specimens (two outliers and four *T. georgianus* reclassified by CVA as other *Taphozous* species), all fossil specimens clustered tightly within the *T. georgianus* 95% ellipse (Fig. 12). However, this PCA used dental variables as well as cranial, for which data is mostly absent for the fossil specimens (e.g. the ten variables with the highest loadings were not represented in the fossils).

**Figure 13:**
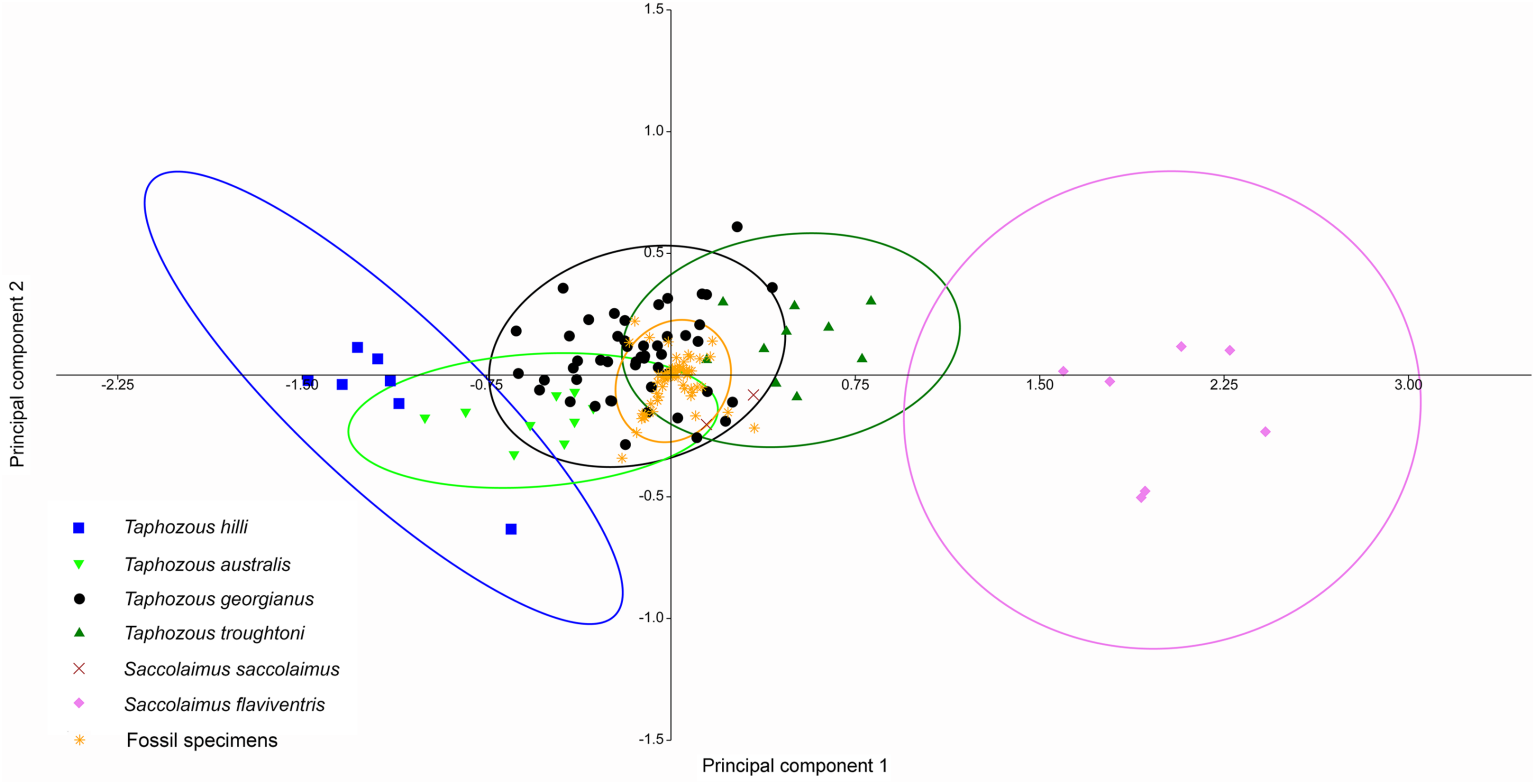
PCA of fossil and extant emballonurids, using only dental data. Most fossil specimens cluster close to the *T. georgianus* centroid, although some also overlap with *T. troughtoni*, *T. australis* and near *S. saccolaimus*. Ellipses are 95% confidence limits. PC1 and PC2 represent 71.6% and 6.3% of variation, respectively.

A PCA was also run using only dental variables for which more than one data point was represented in the fossil sample (Fig. 13). Most fossil specimens clustered close to the *T. georgianus* centroid, although some specimens also overlapped with *T. troughtoni* and *T. australis* ellipses and near *Saccolaimus saccolaimus*. First upper molar width and length had the highest loadings on PC1, separating *Taphozous* species from each other, and *Saccolaimus flaviventris* from all other species.

**Figure 14:**
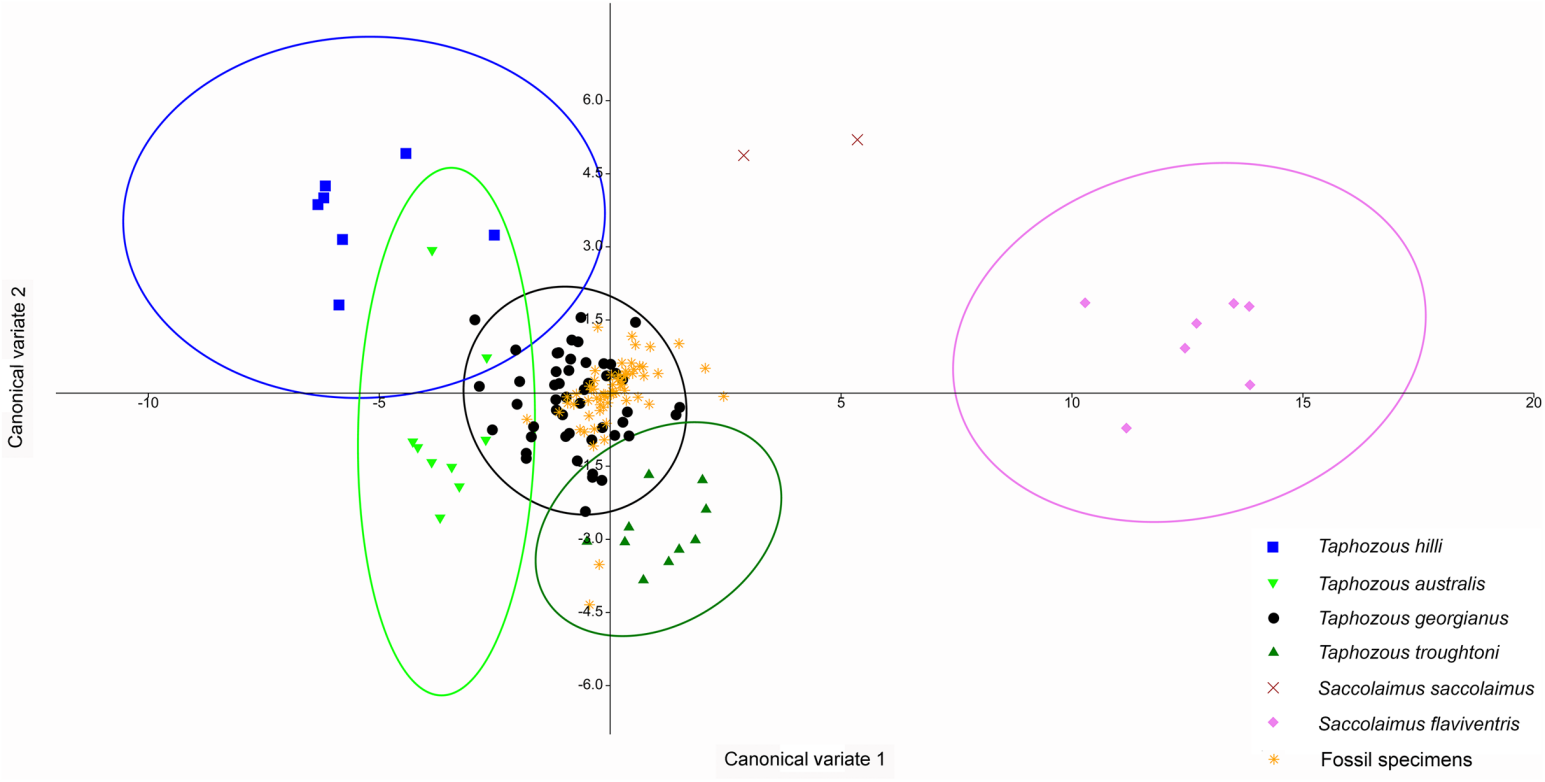
CVA of fossil and extant emballonurids using dental variables only. All but 3 fossil specimens classified as *T. georgianus* (QM F23868, QM F23846 and QM F23845 classified as *T. troughtoni*). Ellipses are 95% confidence limits. Canonical variates 1 and 2 represent 77.4% and 12.2% of variation, respectively.

A CVA performed on the same dataset also indicated the congruity of *T. georgianus* and most fossil specimens, with only three specimens (QM F23868, QM F23846 and QM F23845) being classified as *T. troughtoni* (Fig. 14). Upper first molar length and width were again the greatest contributors to separating species on axis one (77.4% of variation), while upper canine and upper fourth premolar length were important for separating *T. hilli* and *T. troughtoni* from other *Taphozous* species on axis two (12.2% of variation).

**Figure 15:**
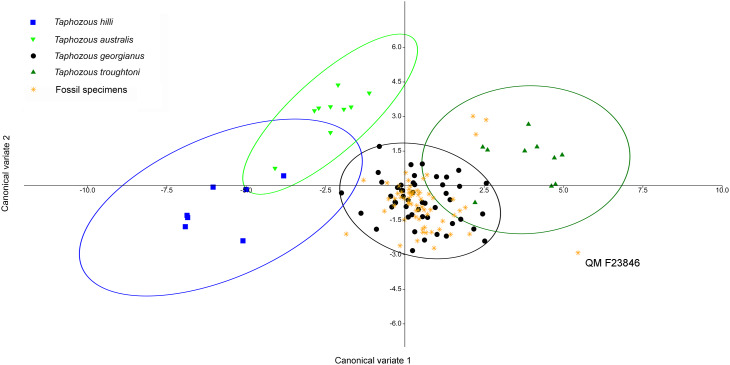
CVA of fossil and extant emballonurids (*Saccolaimus* spp. removed), using dental variables only. QM F23846 is isolated from all *Taphozous* spp. Ellipses are 95% confidence limits. Canonical variates 1 and 2 represent 72.3% and 20.8% of variation, respectively.

*Saccolaimus* species were isolated from each other, as well as all *Taphozous* and the fossil specimens. Removal of *Saccolaimus* spp. from the CVA increased separation of *Taphozous* species (Fig. 15; for example *T. australis* partially overlaps only with *T. hilli*). Lower canine length and upper first molar length were the largest contributors to species differentiation on axis one (72.3% of variation), while upper canine length and upper first molar width were important for separating *T. australis* from other *Taphozous* species on axis two (20.8% of variation). One specimen, previously classified as *T. troughtoni* (QM F23846), was isolated from *Taphozous* species (Fig. 15). These results therefore support the morphological analysis conclusion that QM F23846 may represent a new species of *Saccolaimus*.

## Discussion

### Quantitative assessment of species boundaries in extant Australian emballonurids

Chimimba & Kitchener (1991) used morphological systematics, protein electrophoresis and immunology to separate the eight extant Australian emballonurid species. The examination here of six emballonurid species (*T. kapalgensis* and *S. mixtus* having been omitted due to insufficient data) supports the taxonomic validity of species proposed by Chimimba & Kitchener (1991).

Although Principal Components Analysis (PCA) does not test hypotheses, it can help visualise natural groupings that correspond to interspecific differences and/or similarities in multivariate data (Hammer & Harper, 2006). In the PCA of six extant Australian taxa, *Saccolaimus flaviventris* had the greatest magnitude of separation from other emballonurid species, while *S. saccolaimus* grouped closer to all species of *Taphozous* (being separated only by the less significant PC2). Although the 95 percent ellipses at least partially overlapped for all species of *Taphozous*, the centroids for *T. hilli, T. georgianus* and *T. troughtoni* were separated on PC1, while *T. georgianus* and *T. australis* separated on PC2. This reflects a close morphological similarity among most Australian *Taphozous* species, with absolute size (PC1) contributing most to the separation. In all analyses of modern taxa here, *S. flaviventris* also separates from other emballonurid species mostly on the basis of its much larger size. Chimimba & Kitchener (1991) recognised that *S. flaviventris* and *S. saccolaimus* are morphologically similar, while also being morphologically distinct from all other Australian emballonurids.

Canonical Variate Analysis (CVA) of combined cranial and dental characters provided the best means of discriminating all Australian emballonurid species examined. However, the most significant discriminators of emballonurid species were primarily cranial. Characters relating to skull and dentary length (BL, GL and DL) and width of the posterior nose-shield, mastoid and zygomatic arch (PNS, MW and ZW) were the most important for distinguishing: (1) *Saccolaimus* species from each other; (2) *Saccolaimus* from *Taphozous*; and (3) *T. troughtoni* from other *Taphozous* species. For differentiation of *Taphozous* species, rostrum length (ROL) and, inversely, distance outside promontorium (OP) and least interorbital width (LOW) were also important variables. At one end of the morphocline, *T. troughtoni* is characterised, for example, by a relatively long and narrow snout, while *T. hilli* possesses a shorter and wider rostrum.

The CVA results largely reflect size variation between species, with the highest loadings supporting the conclusions of other studies that have investigated size variation (Freeman, 1981; Chimimba & Kitchener, 1991). This is not surprising because, for instance, the mean maxillary tooth row length in *T. hilli* (8.89 mm), *T. georgianus* (10.29 mm) and *S. flaviventris* (12.38 mm) appears to reflect a size cline. It has been suggested that such clines (possibly the result of character displacement to reduce competition between sympatric congeners) can result from resource partitioning among otherwise morphologically similar and closely related species of insect-eating bats (Freeman, 1981; Chimimba & Kitchener, 1991). Evidence for sexual dimorphism could not be found, at least in *T. georgianus*, supporting the findings of both Chimimba & Kitchener (1991) and Carpenter, McKean & Richards (1978) that cave-dwelling bat species display less pronounced sexual dimorphism than their forest-dwelling counterparts.

Canonical Variate Analysis confirmed that at least 14 of the 20 extant *Taphozous georgianus* specimens included here, but collected east of Mount Isa, were correctly identified, with the range extending north as far Chillagoe and east to Rockhampton, Queensland. This is consistent with some previously proposed distributions for this species (Chimimba & Kitchener, 1991). Two specimens, AM M27892 and AM M8550, were consistently outliers and re-classified as *T. hilli* and *T. troughtoni* respectively. AM M27892 is from the Alligator River in the Northern Territory, which is consistent with the central and western Australian distribution for *T. hilli*, albeit a northern outlier. AM M8550 is from Barry Cave, in the Northern Territory, approximately 150 km west of Camooweal. This supports the range extension for *T. troughtoni* suggested by Reardon & Thomson (2002). CVA also indicated that at least one other specimen identified as *T. georgianus* from east of Mount Isa, AMNH107770, may also be *T. hilli*. This specimen was re-classified as *T. hilli* primarily based on a very large occipital region (OP) and relatively long M^1^. However, AMNH10770 is from Pentland, near Charters Towers, Queensland, which would extend the known distribution of *T. hilli* east by several hundred kilometres. CVA results also placed this specimen close to the *T. australis* cluster. This specimen should be re-examined to confirm the identification as *T. hilli*.

A further three specimens, originally identified as *T. georgianus* from east of Mount Isa, were re-classified in the CVA as *T. australis*: AMNH183557 and AMNH66145 from Chillagoe, Qld and AM M6024 from Morinish, near Rockhampton, Qld, sites within 150 km of the east coast. *Taphozous australis* is usually described as having a coastal distribution (Duncan, Baker & Montgomery, 1999). However, records are known from inland localities, including central Cape York (Atlas of Living Australia, 2020), and it is feasible that the three specimens are *T. australis*.

For modern emballonurid taxa, intraspecific variation in the metric characters, as measured by the Coefficient of Variation (CV), was generally low (falling within that expected for single species samples (Cope & Lacy, 1992)). However, the dimensions of M^3^ and molar cristid characters were particularly variable within species compared to all other craniodental measurements (Supplemental Data S3 (Table S3.2)). High variability in posterior upper molar metrics has been identified in many mammal groups including, for example, canids (Gingerich & Winkler, 1979), viverravids (Gingerich & Winkler, 1985) and primates (Gingerich & Schoeninger, 1979; Morita, Morimoto & Ohshima, 2016). Polly (1998), however, cautioned that a correlation between CV and size may artificially inflate apparent variability, although this could not be confirmed for carnivores in subsequent studies (Dayan, Wool & Simberloff, 2002; Meiri, Dayan & Simberloff, 2005). Similarly, Wolsan et al. (2015) found that hypotheses concerning developmental fields, the timing of tooth formation, occlusal complexity and sexually dimorphic hormonal activity, could not explain size variation along the pinniped toothrow. Until such relationships are examined in emballonurids, the M^3^ should be considered unreliable for discriminating between Australian species.

The similarity in size and morphology between *T. georgianus* and *T. troughtoni* precludes separation of these species based on qualitative and quantitative analyses of dental characters alone. However, the inclusion of cranial characters increased taxonomic resolution. It is clear that cranial material is essential for the correct identification of these two often-confused species.

### Qualitative and quantitative assessment of fossil emballonurids from Rackham’s Roost Site

The results indicate that CV values for M_3_ show the most variability of all measurements (Supplemental Data S3 (Table S3.3)). This reflects variation in both width and length of M_3_ and, combined with CV values for a suite of other craniodental characters, is consistent with recognition of more than one emballonurid species within the Rackham’s Roost Site fossil sample. Qualitative and quantitative analyses indicate the presence of three emballonurid taxa: *Taphozous georgianus, T. troughtoni* and *Saccolaimus* sp. These are the first emballonurids described from the Australian fossil record.

Although generally larger, fossil specimens retained morphology typically found in species of *Taphozous* rather than *Saccolaimus* (see “Systematics”). Multivariate analyses could not differentiate most fossil specimens from *T. georgianus*. Two specimens were identified as *T. troughtoni* based on morphometrics of P^4^ and, to give greater confidence, this assignment should be re-examined once further material becomes available.

The large number of *T. georgianus* specimens (*n* = 59), relative to only a few *T. troughtoni* (*n* = 2) and *Saccolaimus* sp. (*n* = 1), may reflect actual relative abundance of the three taxa at that time in the immediate area of the fossil site. Alternatively, it could reflect differences in the proximity and/or susceptibility to predation by the cave-dwelling Ghost Bat *Macroderma gigas* (see “Palaeoecology” below).

The occurrence of *Saccolaimus* sp. (QM F23846) in the Rackham’s Roost assemblage indicates that both genera of Australian emballonurids were present by at least the early Pleistocene. Overall molar dimensions in the fossil taxon differ significantly from the smaller *S. saccolaimus* and *S. mixtus*, with other morphological differences from *S. flaviventris* including the presence of a paracingulum, a paraloph, a well-developed parastyle and a depression between the protocone and heel.

Additional comparative specimens of *Saccolaimus* species are required for quantitative analysis, together with more fossil material, before the identity of the fossil species can be confirmed. However, morphometric analyses presented here indicate that it is unlikely to be *S. saccolaimus* or *S. flaviventris*. The restriction today of both *S. saccolaimus* and *S. mixtus* to north and north-eastern coastal Australia is associated with their habitat preference for tropical forests along the coastal fringe (Churchill, 2009). The absence of wet forest at Riversleigh during the early Pleistocene (Archer, Hand & Godthelp, 2000), also weighs against the Rackham’s Roost *Saccolaimus* representing either of these species.

## Palaeoecology

The fragmentary nature of most of the Rackham’s Roost Site fossils markedly contrasts with that of the majority of Riversleigh’s late Oligocene to Holocene vertebrate fossils which more typically consist of well-preserved material including cranial material, complete dentaries, tooth rows and postcranial elements (Archer, Hand & Godthelp, 2000; Hand, 2012). Based on taphonomic comparisons with the distinctive prey remains of modern carnivorous ghost bats (*M. gigas*) in northern Australia (Toop, 1985; Guppy & Coles, 1983; Schulz, 1986; Hand, 1996; Boles, 1999), the Rackham’s Roost Site fossil assemblage has been interpreted to mostly consist of the remains of small vertebrate prey brought into the original cave by that species (Archer, Hand & Godthelp, 2000; Hand, 1996; Godthelp, 1997; Boles, 1998; Nguyen, Hand & Archer, 2016). Frogs, lizards, birds, small marsupials such as dasyurids, small possums and peramelids, and at least 12 species of native murids including species of *Pseudomys*, *Leggadina* and *Zyzomys* have been identified among the fossil prey remains (Hand, 1996; Godthelp, 1997; Boles, 1998; Klinkhamer & Godthelp, 2015; Nguyen, Hand & Archer, 2016).

The original Rackham’s Roost Site cave is thought to have also provided shelter for a variety of other animals, such as snakes, pseudocheirids, potoroids (*Bettongia* sp.), several macropodids including at least two species of *Macropus* and one of *Protemnodon* (Archer, Hand & Godthelp, 2000), and other bats. Most Australian *Taphozous* species are cave-dwelling and it is likely that the fossil *Taphozous* species lived in or around the Rackham’s Roost Site cave. Australian *Saccolaimus* species on the other hand are typically tree roosting bats (Churchill, 2009), and are high, fast-flyers that would be unlikely to be captured by *Macroderma*. In Asia, roosts in rocky habitats have been reported for *Saccolaimus* (Francis, 2019), and this may have been also the case for this early Pleistocene species in northern Australia. Until more material of *Saccolaimus* sp. is known, it is not possible to determine what its roosting habits may have been.

*Macroderma gigas* is known to kill and eat vertebrates up to the size of a pigeon, and their prey includes other bats (Douglas, 1967; Claramunt et al., 2019; Start, McKenzie & Bullen, 2019). Several *Taphozous* fossils examined in the present study (including AR21229; Fig. 2A) show depressed fractures and damage consistent with predation by *M. gigas* (Guppy & Coles, 1983; Hand, 1995, 1996; Boles, 1999), as well as at least one specimen (AR21220; Fig. 2K) that completely lacks enamel and is interpreted here to have been digested by *M. gigas* (see also Hand, 1996).

The Rackham’s Roost bat fauna includes a mixture of 11 extant and extinct bat species. Extinct taxa include *Hipposideros winsburyorum* (Hand & Godthelp, 1999) and *Me. richardsi* (Hand, 1995). Extant species include *Macroderma gigas* and *Rhinonicteris aurantia* (Archer, Hand & Godthelp, 2000; Hand, 1996), as well as the two *Taphozous* species and one *Saccolaimus* sp. identified here. The oldest Australian representatives of modern vespertilionid genera (*Chalinolobus* and *Scotorepens*) and the last members of the once ubiquitous *Brachipposideros* radiation also occur in this deposit (Hand, 2006, 2012). It is not yet clear whether *Saccolaimus* sp. is an extinct or extant species but, collectively, the Rackham’s Roost Site bat taxa appear to represent a link between bat faunas of the Oligo-Miocene and the present (Hand et al., 2013).

From the fauna represented in the deposit (Archer, Hand & Godthelp, 2000; Travouillon et al., 2006) and more general information about Australian early Pleistocene climate (Martin, 2006), the palaeohabitat around Riversleigh’s Rackham Roost Site is thought to have been similar to open woodland, probably not very different from that found in the area today, and indeed around Mount Isa where the modern distributions of *T. georgianus* and *T. troughtoni* overlap.

A study of the endemic Australian rhinonycterid *Rhinonicteris aurantia*, focusing on the isolation of populations across northern Australia, remarked on the general patterns of divergence in several groups of animals caused by the Great Sandy Desert (Armstrong, 2002). When the aridification of Australia began in the late Cenozoic, the persistence of geographically isolated remnants of less-arid habitat led to allopatric populations of taxa adapted to relatively more mesic environments (Armstrong, 2002). Ancestral populations of *T. georgianus* and *T. troughtoni* were likely isolated in northern Australia because of a process of desertification, and reproductive isolation that developed in allopatry was reinforced when they eventually came back into contact in western Queensland. The confusion for identifying these two species is the result of them being sister taxa, and their similar size outside the area of contact.

## Conclusions

The Rackham’s Roost Site bat fossils reported here indicate that three emballonurid species were living in the Riversleigh area of north-western Queensland in the early Pleistocene. Among the fossil remains, the modern species *Taphozous georgianus* is best represented, but the extant *T. troughtoni* is also recorded, as is a single specimen here referred to the genus *Saccolaimus*. In this study, multivariate analyses of combined cranial and dental characters best distinguished between Australian emballonurids, although species could also be differentiated using dental variables alone. Our results suggest that the modern distribution of *T. georgianus* may extend from the Pilbara region in Western Australia east to Rockhampton, Queensland, while the range of *T. troughtoni* appears to extend east of Mount Isa, possibly to the east coast, and west to within the Northern Territory. The results also suggest a range extension for *T. australis* from coastal Queensland to at least inland Chillagoe and south to Rockhampton.

## Supplemental Information

10.7717/peerj.10857/supp-1 Supplemental Information 1 Specimens used in the study.

10.7717/peerj.10857/supp-2 Supplemental Information 2 Modern emballonurid skull photographs.

10.7717/peerj.10857/supp-3 Supplemental Information 3 Univariate and multivariate statistics for extant and fossil specimens.

10.7717/peerj.10857/supp-4 Supplemental Information 4 Raw measurement data for all specimens.

## Additional Information and Declarations

### Data Availability

The following information was supplied regarding data availability: Raw measurements for all extant and fossil specimens, which provided the basis for all morphometric analyses, are available in the Supplemental Files. Fossil specimens are from the Queensland Museum fossil collection, Brisbane, Australia (prefix QM F); University of NSW vertebrate fossil collection, Sydney, Australia (prefix AR); American Museum of Natural History, New York, USA (prefix AMNH); Australian Museum mammal collection, Sydney, Australia (prefix AM M); and the Museum and Art Gallery of the Northern Territory, Darwin, Australia (prefix U).

## References

[ref-1] Anderson MJ (2006). Distance-based tests for homogeneity of multivariate dispersions. Biometrics.

[ref-2] Archer M, Clayton G, Hand SJ, Archer M, Clayton G (1984). A checklist of Australasian fossil mammals. Vertebrate Zoogeography and Evolution in Australasia.

[ref-3] Archer M, Hand SJ, Godthelp H (2000). Australia’s lost world: Riversleigh, World Heritage Site.

[ref-4] Armstrong KN (2002). Morphometric divergence among populations of *Rhinonicteris aurantius* (Chiroptera : Hipposideridae) in northern Australia. Australian Journal of Zoology.

[ref-5] Armstrong KD (2020). *Taphozous hilli*—the IUCN Red List of Threatened Species. https://dx.doi.org/10.2305/IUCN.UK.2020-2.RLTS.T21457A22110905.en.

[ref-6] Armstrong K, Reardon T (2017). *Taphozous georgianus*—the IUCN Red List of Threatened Species. https://dx.doi.org/10.2305/IUCN.UK.2017-2.RLTS.T21454A22111763.en.

[ref-7] Armstrong K, Reardon T, Woinarski J, Burbidge AA (2017). *Taphozous troughtoni*—the IUCN Red List of Threatened Species. https://dx.doi.org/10.2305/IUCN.UK.2017-2.RLTS.T21466A22109564.en.

[ref-8] Atlas of Living Australia (2020). Australia’s biodiversity data. http://www.ala.org.au.

[ref-9] Boles WE (1998). A Budgerigar *Melopsittacus undulatus* from the Pliocene of Riversleigh, North-western Queensland. Emu—Austral Ornithology.

[ref-10] Boles WE (1999). Avian prey of the Australian Ghost Bat *Macroderma gigas* (Microchiroptera: Megadermatidae): prey characteristics and damage from predation. Australian Zoologist.

[ref-11] Bonaccorso F, Wilson DE, Mittermeier RA (2019). Family emballonuridae (Sheath-tailed Bats). Handbook of the Mammals of the World.

[ref-12] Carpenter SM, McKean JL, Richards GC (1978). Multivariate morphometric analysis of *Eptesicus* (Mammalia : Chiroptera) in Australia. Australian Journal of Zoology.

[ref-13] Chimimba T, Kitchener J (1991). A systematic revision of Australian Emballonuridae (Mammalia: Chiroptera). Records of the Western Australian Museum.

[ref-14] Churchill S (2009). Australian bats.

[ref-15] Claramunt AMA, White NE, Bunce M, O’Connell M, Bullen RD, Mawson PR (2019). Determination of the diet of the ghost bat (*Macroderma gigas*) in the Pilbara region of Western Australia from dried prey remains and DNA metabarcoding. Australian Journal of Zoology.

[ref-16] Cope D, Lacy MG (1992). Falsification of a single species hypothesis using the coefficient of variation: a simulation approach. American Journal of Physical Anthropology.

[ref-64] Cope DA, Lacy MG (1995). Comparative application of the coefficient of variation and range-based statistics for assessing the taxonomic composition of fossil samples. Journal of Human Evolution.

[ref-17] Davis JC (1986). Statistics and data analysis in geology.

[ref-18] Dayan T, Wool D, Simberloff D (2002). Variation and covariation of skulls and teeth: modern carnivores and the interpretation of fossil mammals. Paleobiology.

[ref-19] Douglas AM (1967). The natural history of the Ghost Bat, *Macroderma gigas* (Microchiroptera, Megadermatidae), in Western Australia. Western Australian Naturalist.

[ref-20] Duncan A, Baker GB, Montgomery N (1999). The action plan for Australian bats.

[ref-21] Environment Australia (2000). Revision of the interim biogeographic regionalisation of Australia (IBRA) and the development of version 5.1—summary report.

[ref-22] Francis C (2019). Field guide to the mammals of South-east Asia.

[ref-23] Freeman PW (1981). A multivariate study of the family Molossidae (Mammalia: Chiroptera), morphology, ecology, evolution. Fieldiana Zoology.

[ref-24] Gingerich PD, Schoeninger MJ (1979). Patterns of tooth size variability in the dentition of primates. American Journal of Physical Anthropology.

[ref-25] Gingerich PD, Winkler DA (1979). Patterns of variation and correlation in the dentition of the Red Fox, *Vulpes vulpes*. Journal of Mammalogy.

[ref-26] Gingerich PD, Winkler DA (1985). Systematics of Paleocene Viverravidae (Mammalia, Carnivora) in the Bighorn Basin and Clark’s Fork Basin. Wyoming Contributions from the Museum of Paleontology, The University of Michigan.

[ref-27] Godthelp H (1997). *Zyzomys rackhami* sp. nov. (Rodentia, Muridae) a rockrat from Pliocene Rackham’s Roost Site, Riversleigh, northwestern Queensland. Memoirs of the Queensland Museum.

[ref-28] Guppy A, Coles R (1983). Feeding behavior of the Australian ghost bat, *Macroderma gigas* (Chiroptera: Megadermatidae) in captivity. Australian Mammalogy.

[ref-29] Hall L, Thomson B, Richards G (2008). Taphozous australis—the IUCN Red List of Threatened Species 2008: e.T21452A9278937. https://dx.doi.org/10.2305/IUCN.UK.2008.RLTS.T21452A9278937.en.

[ref-30] Hammer O, Harper DAT (2006). Paleontological data analysis.

[ref-31] Hammer O, Harper DAT, Ryan PD (2001). PAST: paleontological statistics software package for education and data analysis. Palaeontologia Electronica.

[ref-32] Hand SJ (1985). New Miocene megadermatids (Chiroptera: Megadermatidae) from Australia with comments on megadermatid phylogenetics. Australian Mammalogy.

[ref-33] Hand SJ (1995). First record of the genus Megaderma Geoffroy (Microchiroptera: Megaderma) from Australia. Palaeovertebrata.

[ref-34] Hand SJ (1996). New miocene and pliocene megadermatids (Mammalia, Microchiroptera) from Australia, with comments on broader aspects of megadermatid evolution. Geobios.

[ref-35] Hand SJ, Merrick JR, Archer M, Hickey GM, Lee MSY (2006). Bat beginnings and biogeography: the Australian record. Evolution and Biogeography of Australian Vertebrates.

[ref-36] Hand SJ, Lunney D, Hutchings P (2012). Australian bats: differential responses to Cenozoic climate change. Wildlife and Climate Change: Towards Robust Conservation Strategies for Australian Fauna.

[ref-37] Hand SJ, Black KH, Archer M, Godthelp H, Creaser P, Jell PA (2013). Riversleigh world heritage area. Geology of Queensland.

[ref-38] Hand SJ, Godthelp H (1999). First Australian Pliocene species of Hipposideros (Microchiroptera: Hipposideridae). Records of the Western Australian Museum.

[ref-39] Harper DAT, Owen AW, Harper DAT (1999). Quantitative and morphometric methods in taxonomy. Numerical Paleobiology: Computer-based Modelling and Analysis of Fossils and their Distributions.

[ref-42] Klinkhamer AJ, Godthelp H (2015). Two new species of fossil *Leggadina* (Rodentia: Muridae) from Northwestern Queensland. PeerJ.

[ref-43] Martin H (2006). Cenozoic climatic change and the development of the arid vegetation in Australia. Journal of Arid Environments.

[ref-44] Meiri S, Dayan T, Simberloff D (2005). Variability and correlations in carnivore crania and dentition. Functional Ecology.

[ref-45] Morita W, Morimoto N, Ohshima H (2016). Exploring metameric variation in human molars: a morphological study using morphometric mapping. Journal of Anatomy.

[ref-46] Nguyen JMT, Hand SJ, Archer M (2016). The Late Cenozoic passerine avifauna from Rackham’s Roost Site, Riversleigh, Australia. Records of the Australian Museum.

[ref-47] Plavcan JM, Cope DA (2001). Metric variation and species recognition in the fossil record. Evolutionary Anthropology.

[ref-48] Polly PD (1998). Variability in mammalian dentitions: size-related bias in the coefficient of variation. Biological Journal of the Linnean Society.

[ref-49] R Core Team (2017). R: a language and environment for statistical computing.

[ref-50] Reardon T, Thomson B (2002). Taxonomy and conservation status of Troughton’s sheathtail bat (Taphozous troughtoni).

[ref-51] Schulz M (1986). Vertebrate prey of the ghost bat, *Macroderma gigas*, at Pine Creek, Northern Territory. Macroderma.

[ref-52] Simpson GG, Roe A, Lewontin RC (1960). Quantitative zoology.

[ref-53] Smith T, Habersetzer J, Simmons NB, Gunnell GF, Gunnel GF, Simmons NB (2012). Systematics and paleobiogeography of early bats. Evolutionary History of Bats.

[ref-54] Start AN, McKenzie NL, Bullen RD (2019). Notes on bats in the diets of Ghost Bats (*Macroderma gigas*: Megadermatidae) in the Pilbara region of Western Australia. Records of the Western Australian Museum.

[ref-55] Storch G, Sigé B, Habersetzer J (2002). *Tachypteron franzeni* n. gen., n. sp., earliest emballonurid bat from the Middle Eocene of Messel (Mammalia, Chiroptera). Paläontologische Zeitschrift.

[ref-56] Tate GHH (1952). Mammals of Cape York Peninsula, with notes on the occurrence of rain forest in Queensland. Bulletin of the American Museum of Natural History.

[ref-57] Temminck CJ (1838). Taphozoüs Saccolaimus. Tijdschrift voor natuurlijke geschiedenis en physiologie.

[ref-58] Thomas O (1915). Notes on Taphozous and Saccolaimus. Journal of the Bombay Natural History Society.

[ref-59] Thomson B, Pavey C, Reardon T (2001). National recovery plan for cave-dwelling bats, Rhinolophus philippinensis, Hipposideros semoni and Taphozous troughtoni 2001–2005.

[ref-60] Toop J (1985). Habitat requirements, survival strategies and ecology of the ghost bat *Macroderma gigas* Dobson (Microchiroptera, Megadermatidae) in central coastal Queensland. Macroderma.

[ref-61] Travouillon KJ, Archer M, Hand SJ, Godthelp H (2006). Multivariate analyses of Cenozoic mammalian faunas from Riversleigh, northwestern Queensland. Alcheringa.

[ref-62] Wolsan M, Suzuki S, Asahara M, Motokawa M (2015). Tooth size variation in pinniped dentitions. PLOS ONE.

[ref-63] Woodhead J, Hand SJ, Archer M, Graham I, Sniderman K, Arena DA, Black KH, Godthelp H, Creaser P, Price E (2016). Developing a radiometrically-dated chronologic sequence for Neogene biotic change in Australia, from the Riversleigh World Heritage Area of Queensland. Gondwana Research.

