# Supplementary material for: Sheath-tailed bats (Chiroptera: Emballonuridae) from the early Pleistocene Rackham’s Roost Site, Riversleigh World Heritage Area, and the distribution of northern Australian emballonurid species
Source: PeerJ. 2021 Feb 25;9:e10857. doi: 10.7717/peerj.10857 (PMC7916536; doi:10.7717/peerj.10857)
Supplement: Supplemental Information 1 [file peerj-09-10857-s001.docx]

**Supplemental Data S1**

**Complete list of modern and fossil specimens used for the study**

# Table S1.1: Complete list of modern specimens used in this study

| **ID** | **Specimen Type** | **Location** | **SEX** |
| --- | --- | --- | --- |
| AM M3509 | *Taphozous georgianus* | Dunrobin, QLD | M |
| AM M6024 | *Taphozous georgianus* | Morinish, QLD | M |
| AM M8542 | *Taphozous georgianus** | Louie Ck, Adels Grove, QLD | M |
| AM M8543 | *Taphozous georgianus** | Louie Ck, Adels Grove, QLD | M |
| AM M8544 | *Taphozous georgianus** | Louie Ck, Adels Grove, QLD | F |
| AM M8545 | *Taphozous georgianus** | Louie Ck, Adels Grove, QLD | F |
| AM M8546 | *Taphozous georgianus** | Louie Ck, Adels Grove, QLD | F |
| AM M8547 | *Taphozous georgianus** | Louie Ck, Adels Grove, QLD | F |
| AM M8548 | *Taphozous georgianus** | Louie Ck, Adels Grove, QLD | M |
| AM M8549 | *Taphozous georgianus** | Louie Ck, Adels Grove, QLD | F |
| AM M8550 | *Taphozous georgianus* | Barry Cave, Barkley highway, NT | F |
| AM M8551 | *Taphozous georgianus** | Native Bee Mine, Mount Isa, QLD | F |
| AM M8552 | *Taphozous georgianus** | Native Bee Mine, Mount Isa, QLD | M |
| AM M9070 | *Taphozous georgianus** | Riversleigh, QLD | F |
| AM M9491 | *Taphozous georgianus** | Pine Ck, NT | M |
| AM M9878 | *Taphozous georgianus** | Tit Cave, Argyle Downs, WA | M |
| AM M9879 | *Taphozous georgianus** | Tit Cave, Argyle Downs, WA | M |
| AM M9881 | *Taphozous georgianus** | Tit Cave, Argyle Downs, WA | F |
| AM M10352 | *Taphozous georgianus** | 50 km SSW Jabiru, NT | F |
| AM M21429 | *Taphozous georgianus* | Carlton Hill Stat., Kununurm, WA | ? |
| AM M23451 | *Taphozous georgianus** | Riversleigh, QLD | F |
| AM M27892 | *Taphozous georgianus* | Dempilli, Alligator River, NT | F |
| AM M23452 | *Taphozous georgianus** | Riversleigh, QLD | F |
| AM M8201 | *Taphozous hilli* | Ayers Rock, NT | F |
| AM M4424 | *Taphozous australis* | Possession Is, Torres St, QLD | M |
| AM M4422 | *Taphozous australis* | Possession Is, Torres St, QLD | M |
| AM M4419 | *Taphozous australis* | Possession Is, Torres St, QLD | M |
| AM M4413 | *Taphozous australis* | Possession Is, Torres St, QLD | F |
| AM M4416 | *Taphozous australis* | Possession Is, Torres St, QLD | M |
| AM M2397 | *Taphozous australis* | New Guinea | ? |
| AM M2398 | *Taphozous australis* | New Guinea | ? |
| AM M4417 | *Taphozous australis* | Possession Is, Torres St, QLD | M |
| AM M4415 | *Taphozous australis* | Possession Is, Torres St, QLD | M |
| AM M3490 | *Saccolaimus saccolaimus* | Kalutara, Ceylon | F |
| AM M3491 | *Saccolaimus saccolaimus* | Kalutara, Ceylon | M |
| AM M3228 | *Saccolaimus flaviventris* | Moree, NSW | F |
| AM M8190 | *Saccolaimus flaviventris* | Brewarrina, NSW | M |
| AM M5057 | *Saccolaimus flaviventris* | Groote Eylandt, NT | F |
| AM M8583 | *Saccolaimus flaviventris* | Smoke Hills, NT | F |
| AM M21226 | *Saccolaimus flaviventris* | Groote Eylandt, NT | F |
| AMNH107736 | *Taphozous troughtoni* | Quamby, QLD | F |
| AMNH107751 | *Taphozous troughtoni* | Quamby, QLD | M |
| AMNH107740 | *Taphozous troughtoni* | Quamby, QLD | F |
| AMNH107761 | *Taphozous troughtoni* | Quamby, QLD | F |
| AMNH107749 | *Taphozous troughtoni* | Quamby, QLD | M |
| AMNH109263 | *Taphozous troughtoni* | Quamby, QLD | F |
| AMNH109269 | *Taphozous troughtoni* | Pentland, QLD | M |
| AMNH109265 | *Taphozous troughtoni* | Quamby, QLD | F |
| AMNH107750 | *Taphozous troughtoni* | Quamby, QLD | M |
| AMNH162707 | *Taphozous troughtoni* | Rifle Creek, Mt Isa, QLD | M |
| AMNH197182 | *Taphozous georgianus** | Parry Creek, WA | M |
| AMNH183439 | *Taphozous georgianus** | Red Bank Mine, NT | F |
| AMNH109266 | *Taphozous georgianus* | Quamby, QLD | M |
| AMNH183557 | *Taphozous georgianus* | Chillagoe Caves, QLD | F |
| AMNH107752 | *Taphozous georgianus* | Quamby, QLD | F |
| AMNH183440 | *Taphozous georgianus** | Red Bank Mine, NT | F |
| AMNH197194 | *Taphozous georgianus** | Pullcunah Hill, Woodstock, WA | M |
| AMNH183437 | *Taphozous georgianus** | Red Bank Mine, NT | M |
| AMNH107733 | *Taphozous georgianus* | Quamby, QLD | F |
| AMNH183438 | *Taphozous georgianus** | Red Bank Mine, NT | F |
| AMNH160387 | *Taphozous georgianus** | Katherine, NT | M |
| AMNH107770 | *Taphozous georgianus* | Pentland, QLD | F |
| AMNH162706 | *Taphozous georgianus* | Mt. Etna, Rockhampton, QLD | M |
| AMNH107769 | *Taphozous georgianus* | Pentland, QLD | M |
| AMNH107747 | *Taphozous georgianus* | Quamby, QLD | F |
| AMNH109267 | *Taphozous georgianus* | Quamby, QLD | M |
| AMNH66145 | *Taphozous georgianus* | Chillagoe Caves, QLD | M |
| AMNH197199 | *Taphozous georgianus** | Woodstock, WA | F |
| AMNH197560 | *Taphozous georgianus** | Tambrey, WA | M |
| AMNH153516 | *Taphozous georgianus* | QLD | ? |
| AMNH183552 | *Taphozous georgianus* | Mungana Caves, QLD | M |
| AMNH197183 | *Taphozous georgianus** | Mundabullangana, Peawah, WA | F |
| AMNH197177 | *Taphozous georgianus* | Black Elvire River, WA | ? |
| AMNH197175 | *Taphozous georgianus** | Mt Anderson, WA | F |
| AMNH107731 | *Taphozous georgianus* | Quamby, QLD | M |
| AMNH109264 | *Taphozous georgianus* | Quamby, QLD | M |
| AMNH197559 | *Taphozous georgianus** | Inglis Gap, WA | M |
| AMNH197200 | *Taphozous georgianus** | 6 mi. N.E. Yardie Homestead, WA | M |
| AMNH197176 | *Taphozous georgianus** | Black Elvire River, WA | M |
| AMNH107753 | *Taphozous georgianus* | Quamby, QLD | F |
| AMNH107748 | *Taphozous georgianus* | Quamby, QLD | M |
| AMNH107754 | *Taphozous georgianus* | Quamby, QLD | M |
| U3892 | *Taphozous hilli* | Mt. Samuel, WA | ? |
| U3910 | *Taphozous hilli* | Coniston Station, NT | ? |
| U3889 | *Taphozous hilli* | Tennant Ck area, NT | ? |
| U3890 | *Taphozous hilli* | Tennant Ck area, NT | ? |
| U3906 | *Taphozous hilli* | Hermannsburg Stn, NT | ? |
| U3902 | *Taphozous hilli* | Hatches Ck Mine, NT | ? |
| U3828 | *Saccolaimus flaviventris* | Murranji Stock Reserve, NT | ? |
| U3831 | *Saccolaimus flaviventris* | Katherine Gorge National Park, NT | ? |

* - specimens from west of Mount Isa, Qld, used in sexual dimorphism analysis

# Table S1.2: Complete list of fossil specimens used in this study

| **ID** | **Specimen Type** | **Location** |
| --- | --- | --- |
| AR21191 | L C^1^ | Rackham’s Roost Site, Riversleigh WHA, QLD |
| AR21192 | L M_3_ | Rackham’s Roost Site, Riversleigh WHA, QLD |
| AR21193 | L M_2_ | Rackham’s Roost Site, Riversleigh WHA, QLD |
| AR21194 | R M^2^ | Rackham’s Roost Site, Riversleigh WHA, QLD |
| AR21195 | L M^1^ | Rackham’s Roost Site, Riversleigh WHA, QLD |
| AR21196 | L M^2^ | Rackham’s Roost Site, Riversleigh WHA, QLD |
| AR21197 | L M_2_ | Rackham’s Roost Site, Riversleigh WHA, QLD |
| AR21198 | R M^2^ | Rackham’s Roost Site, Riversleigh WHA, QLD |
| AR21199 | R M^2^ | Rackham’s Roost Site, Riversleigh WHA, QLD |
| AR21200 | L M_2_ | Rackham’s Roost Site, Riversleigh WHA, QLD |
| AR21201 | R M^2^ | Rackham’s Roost Site, Riversleigh WHA, QLD |
| AR21202 | R C^1^ | Rackham’s Roost Site, Riversleigh WHA, QLD |
| AR21203 | L C_1_ | Rackham’s Roost Site, Riversleigh WHA, QLD |
| AR21204 | R C^1^ | Rackham’s Roost Site, Riversleigh WHA, QLD |
| AR21205 | R C^1^ | Rackham’s Roost Site, Riversleigh WHA, QLD |
| AR21206 | L M^2^ | Rackham’s Roost Site, Riversleigh WHA, QLD |
| AR21207 | L C^1^ | Rackham’s Roost Site, Riversleigh WHA, QLD |
| AR21208 | R M_2_ | Rackham’s Roost Site, Riversleigh WHA, QLD |
| AR21209 | R M^1^ | Rackham’s Roost Site, Riversleigh WHA, QLD |
| AR21210 | R M_2_ | Rackham’s Roost Site, Riversleigh WHA, QLD |
| AR21211 | R C_1_ | Rackham’s Roost Site, Riversleigh WHA, QLD |
| AR21213 | R C^1^ | Rackham’s Roost Site, Riversleigh WHA, QLD |
| AR21215 | L C^1^ | Rackham’s Roost Site, Riversleigh WHA, QLD |
| AR21216 | L C_1_ | Rackham’s Roost Site, Riversleigh WHA, QLD |
| AR21218 | L C^1^ | Rackham’s Roost Site, Riversleigh WHA, QLD |
| AR21219 | R P_4_ | Rackham’s Roost Site, Riversleigh WHA, QLD |
| AR21220 | L M_1_ | Rackham’s Roost Site, Riversleigh WHA, QLD |
| AR21221 | L M^2^ | Rackham’s Roost Site, Riversleigh WHA, QLD |
| AR21222 | R C^1^ | Rackham’s Roost Site, Riversleigh WHA, QLD |
| AR21223 | L M^2^ | Rackham’s Roost Site, Riversleigh WHA, QLD |
| AR21224 | R M^2^ | Rackham’s Roost Site, Riversleigh WHA, QLD |
| AR21225 | R C_1_ | Rackham’s Roost Site, Riversleigh WHA, QLD |
| AR21226 | L M_3_ | Rackham’s Roost Site, Riversleigh WHA, QLD |
| AR21227 | R C^1^ | Rackham’s Roost Site, Riversleigh WHA, QLD |
| AR21229 | L maxilla fragment with P^4^ – M^1^ | Rackham’s Roost Site, Riversleigh WHA, QLD |
| AR21230 | L M^2^ | Rackham’s Roost Site, Riversleigh WHA, QLD |
| AR21231 | R M^1^ | Rackham’s Roost Site, Riversleigh WHA, QLD |
| AR21232 | R Dentary fragment with M_3_ | Rackham’s Roost Site, Riversleigh WHA, QLD |
| AR21234 | L M^2^ | Rackham’s Roost Site, Riversleigh WHA, QLD |
| AR21235 | L M^1^ | Rackham’s Roost Site, Riversleigh WHA, QLD |
| AR21236 | L M^2^ | Rackham’s Roost Site, Riversleigh WHA, QLD |
| AR21237 | L M_3_ | Rackham’s Roost Site, Riversleigh WHA, QLD |
| AR21238 | L M^2^ | Rackham’s Roost Site, Riversleigh WHA, QLD |
| AR21239 | R C^1^ | Rackham’s Roost Site, Riversleigh WHA, QLD |
| AR21240 | L C^1^ | Rackham’s Roost Site, Riversleigh WHA, QLD |
| AR21242 | R M^1^ | Rackham’s Roost Site, Riversleigh WHA, QLD |
| AR21243 | L Dentary fragment with M_2-3_ |  |
| QM F23628 | R Dentary fragment  with P_4_ – M_3_ | Rackham’s Roost Site, Riversleigh WHA, QLD |
| QM F23840 | L M^1^ | Rackham’s Roost Site, Riversleigh WHA, QLD |
| QM F23843 | L C^1^ | Rackham’s Roost Site, Riversleigh WHA, QLD |
| QM F23844 | R M^1^ | Rackham’s Roost Site, Riversleigh WHA, QLD |
| QM F23845 | R P^4^ | Rackham’s Roost Site, Riversleigh WHA, QLD |
| QM F23846 | L M^2^ | Rackham’s Roost Site, Riversleigh WHA, QLD |
| QM F23847 | L M_2_ | Rackham’s Roost Site, Riversleigh WHA, QLD |
| QM F23848 | R M^1^ | Rackham’s Roost Site, Riversleigh WHA, QLD |
| QM F23849 | R M^2^ | Rackham’s Roost Site, Riversleigh WHA, QLD |
| QM F23853 | L Dentary fragment  with M_2_ – M_3_ | Rackham’s Roost Site, Riversleigh WHA, QLD |
| QM F23854 | L M^1^ | Rackham’s Roost Site, Riversleigh WHA, QLD |
| QM F23857 | L M_3_ | Rackham’s Roost Site, Riversleigh WHA, QLD |
| QM F23865 | L M^1^ | Rackham’s Roost Site, Riversleigh WHA, QLD |
| QM F23866 | L Dentary fragment  with P_4_ – M_1_ | Rackham’s Roost Site, Riversleigh WHA, QLD |
| QM F23867 | L Dentary fragment  with M_2_ – M_3_ | Rackham’s Roost Site, Riversleigh WHA, QLD |
| QM F23868 | L P^4^ | Rackham’s Roost Site, Riversleigh WHA, QLD |
