# Supplementary material for: Sheath-tailed bats (Chiroptera: Emballonuridae) from the early Pleistocene Rackham’s Roost Site, Riversleigh World Heritage Area, and the distribution of northern Australian emballonurid species
Source: PeerJ. 2021 Feb 25;9:e10857. doi: 10.7717/peerj.10857 (PMC7916536; doi:10.7717/peerj.10857)
Supplement: Supplemental Information 2 [file peerj-09-10857-s002.docx]

**Supplemental Data S2**


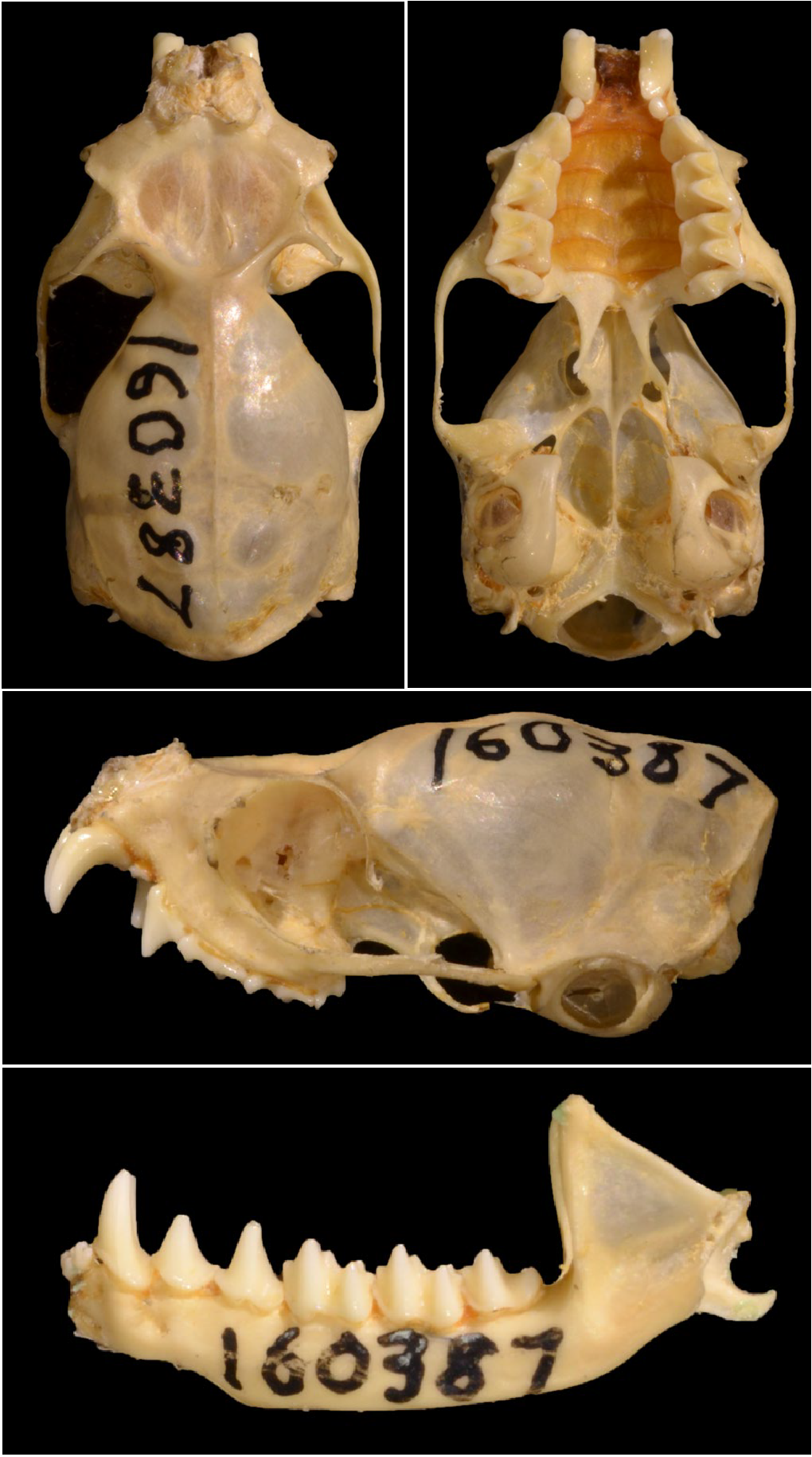


Figure S2.1: *Taphozous georgianus* AMNH160387, Katherine, NT. Scale bar 5 mm.


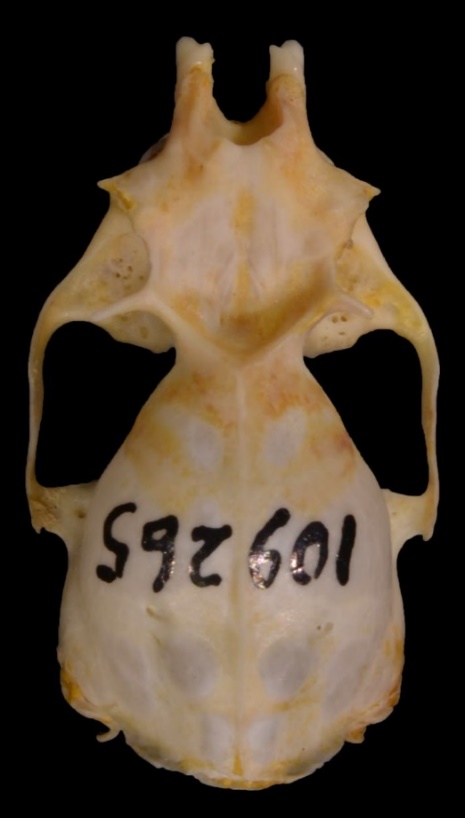

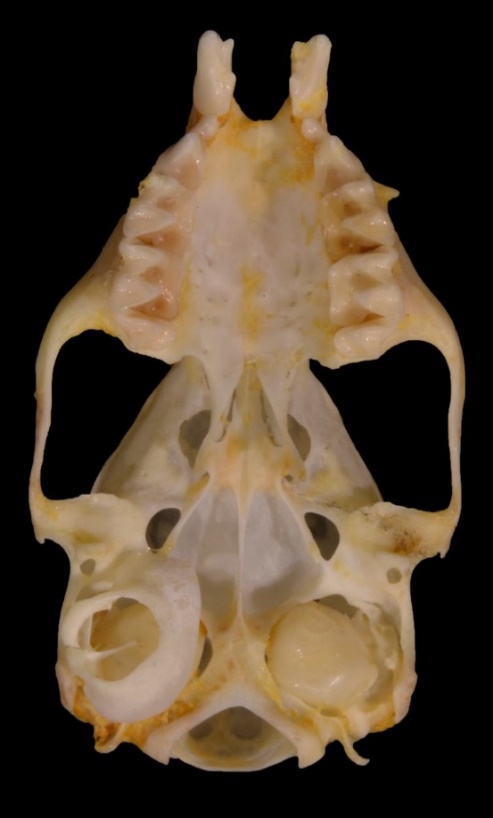

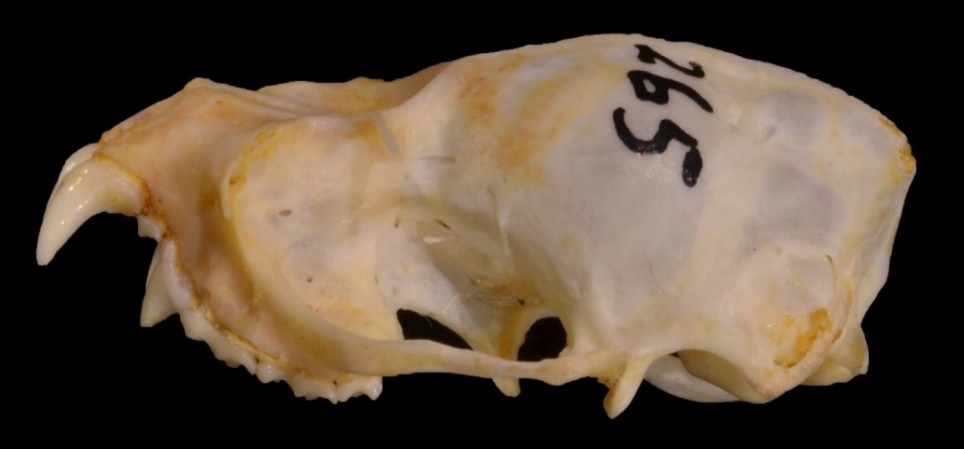

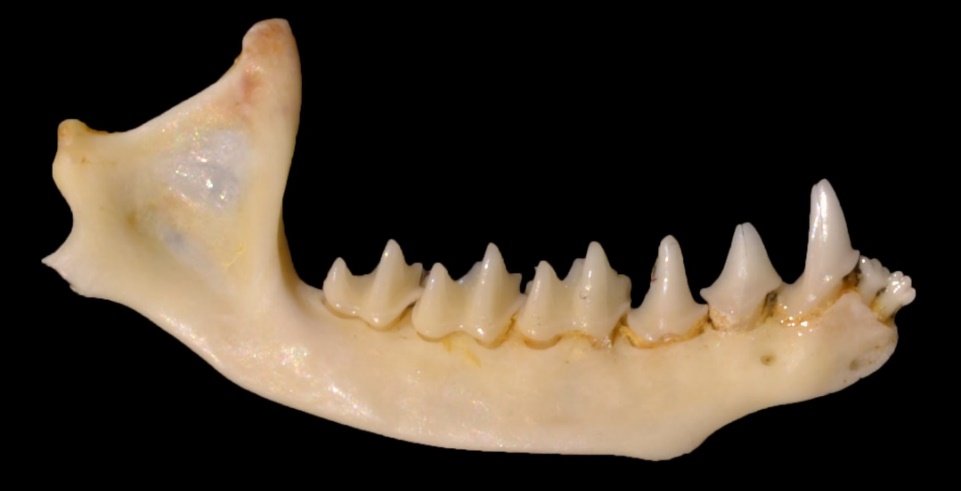


Figure S2.2: *Taphozous troughtoni* AMNH109265, Quamby, QLD. Scale bar 5 mm.


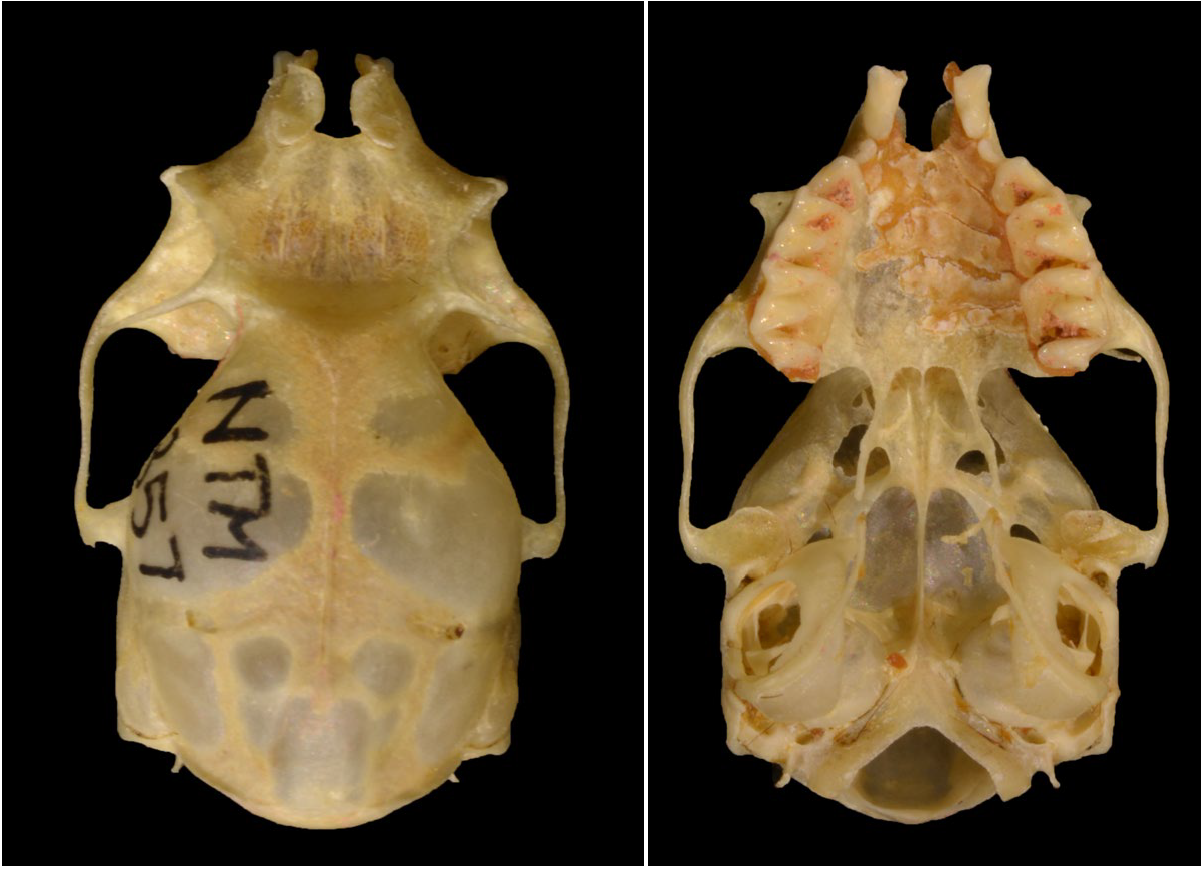

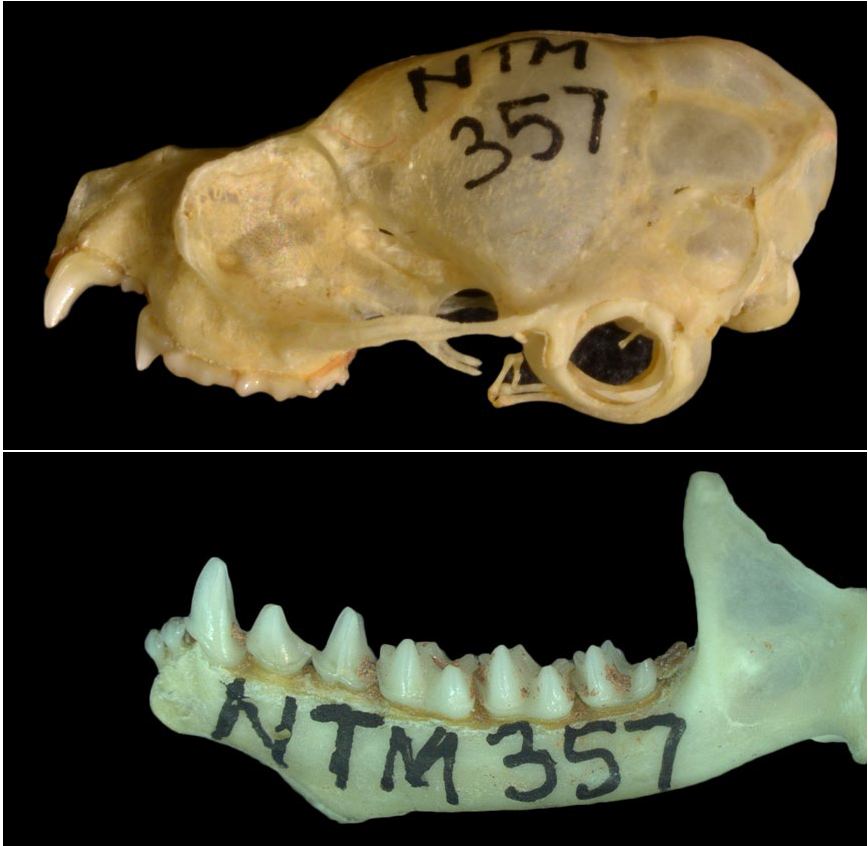


Figure S2.3: *Taphozous hilli* U3889 (NTM 357), Tennant Creek, NT. Scale bar 5 mm.


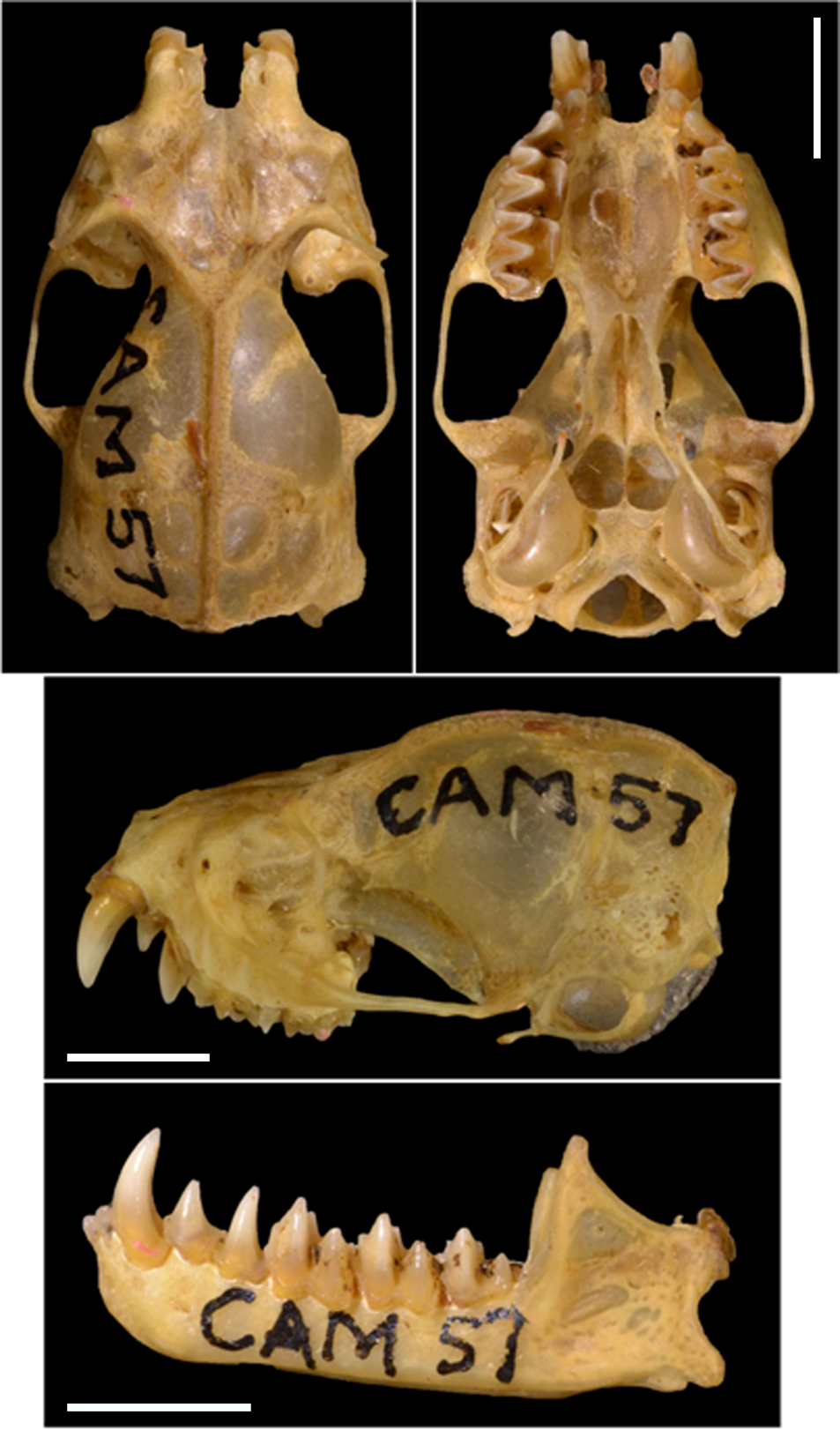


Figure S2.4: *Saccolaimus flaviventris* AM M5057 (CAM 57), Groote Eylandt, NT. Scale bar 5 mm.
