# Supplementary material for: Sheath-tailed bats (Chiroptera: Emballonuridae) from the early Pleistocene Rackham’s Roost Site, Riversleigh World Heritage Area, and the distribution of northern Australian emballonurid species
Source: PeerJ. 2021 Feb 25;9:e10857. doi: 10.7717/peerj.10857 (PMC7916536; doi:10.7717/peerj.10857)
Supplement: Supplemental Information 3 [file peerj-09-10857-s003.docx]

**Supplemental Data S3**

**Univariate and Multivariate Statistical Analysis**


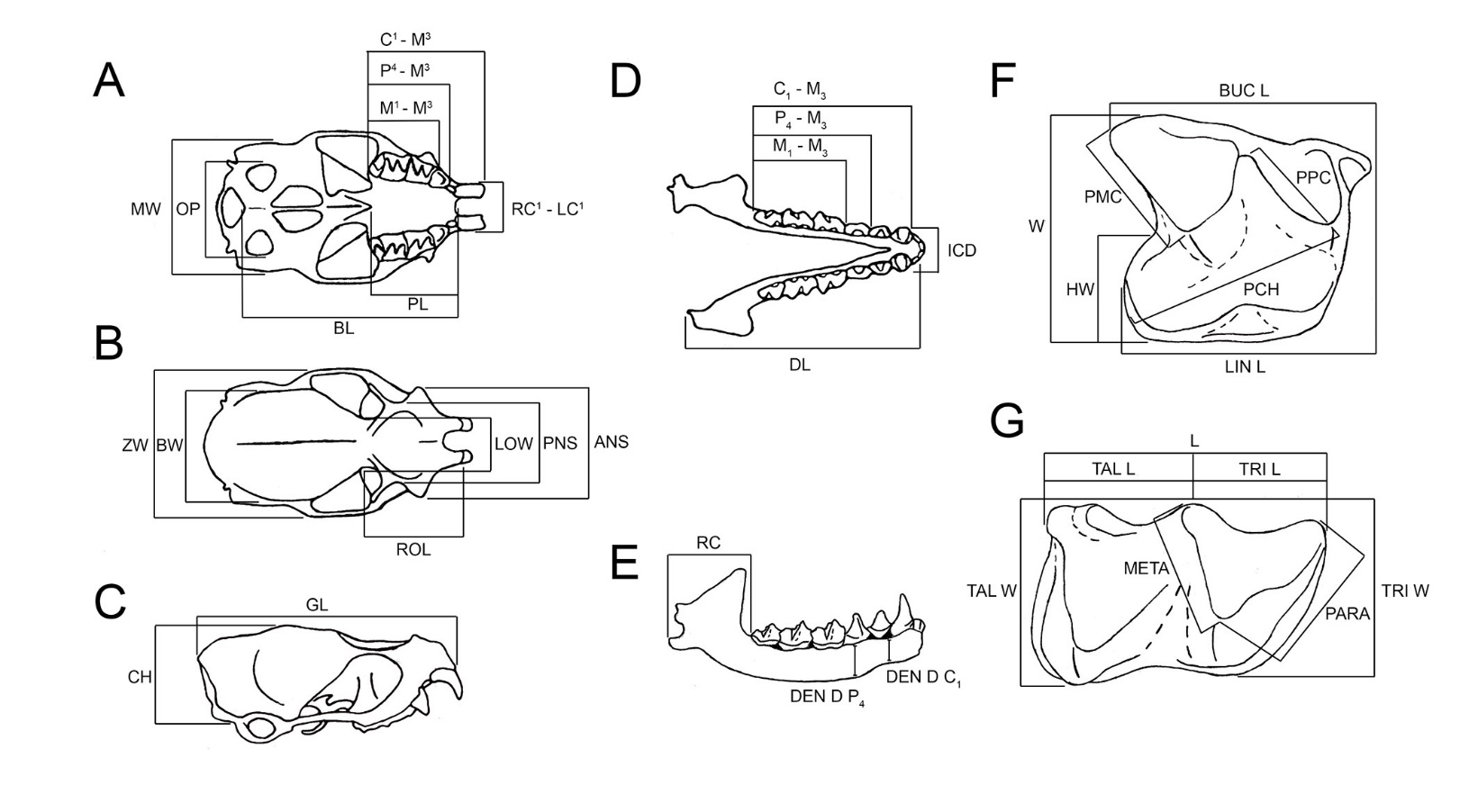


Figure S3.1. Complete m**easurement list referred to in the text for skull, dentary, upper and lower molars (*Taphozous georgianus*), including variables excluded from multivariate analyses as uninformative.** A – skull, ventral view; B – skull, dorsal view; C – skull, lateral view; D – dentaries, dorsal view; E – dentary, buccal view; F – upper molar, occlusal view; G – lower molar, occlusal view. Adapted from Chimimba and Kitchener (1991) and Hand (1985).


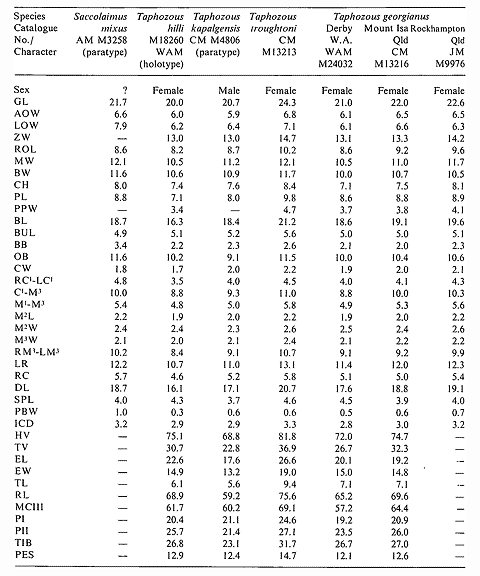
Table S3.1**: Measurements, in mm, for holotypes and paratypes of Australian emballonurids (Source: Chimimba & Kitchener, 1991)**

Table S3.2: **Univariate statistics for modern Australian emballonurids. Abbreviations for parameters: X, Mean; SD, Standard deviation; Mn, Minimum value; Mx, Maximum value; CV, Coefficient of variation; N, Number of specimens. Abbreviations for characters measured given in Glossary, below.**

| **Species** | **Parameters** | **GL** | **MW** | **PL** | **BL** | **OP** | **RC1-LC1** | **C1-M3** | **LOW** | **ZW** | **BW** | **ROL** |
| --- | --- | --- | --- | --- | --- | --- | --- | --- | --- | --- | --- | --- |
| *Taphozous* | X | 21.78 | 11.60 | 8.99 | 18.86 | 9.19 | 4.45 | 10.29 | 4.42 | 13.89 | 10.88 | 8.56 |
| *georgianus* | SD | 0.66 | 0.50 | 0.44 | 0.65 | 0.68 | 0.30 | 0.36 | 0.32 | 0.55 | 0.52 | 0.51 |
| (all specimens) | Mn | 19.82 | 10.30 | 7.94 | 16.87 | 8.10 | 3.02 | 9.13 | 3.33 | 12.38 | 9.84 | 6.98 |
|  | Mx | 24.25 | 12.86 | 10.63 | 21.32 | 11.75 | 5.05 | 11.11 | 5.08 | 15.24 | 12.06 | 10.00 |
|  | CV | **3.05** | **4.30** | **4.93** | **3.47** | **7.36** | **6.83** | **3.51** | **7.15** | **3.93** | **4.80** | **6.00** |
|  | N | 55 | 52 | 55 | 54 | 51 | 50 | 53 | 55 | 49 | 55 | 55 |
| *Taphozous* | X | 21.63 | 11.46 | 9.01 | 18.80 | 9.03 | 4.42 | 10.17 | 4.39 | 13.75 | 10.82 | 8.49 |
| *georgianus* | SD | 0.50 | 0.40 | 0.31 | 0.53 | 0.48 | 0.33 | 0.27 | 0.34 | 0.41 | 0.45 | 0.46 |
| (specimens | Mn | 20.70 | 10.30 | 8.46 | 17.35 | 8.12 | 3.02 | 9.76 | 3.33 | 12.91 | 10.09 | 7.62 |
| West of Mt Isa | Mx | 22.85 | 12.06 | 9.52 | 20.02 | 10.00 | 5.05 | 10.79 | 5.08 | 14.76 | 11.59 | 9.52 |
| only) | CV | **2.33** | **3.46** | **3.43** | **2.82** | **5.36** | **7.55** | **2.66** | **7.69** | **2.95** | **4.17** | **5.39** |
|  | N | 34 | 32 | 34 | 34 | 31 | 31 | 33 | 34 | 32 | 34 | 34 |
| *T. troughtoni* | X | 23.87 | 12.73 | 10.05 | 20.95 | 9.62 | 4.94 | 11.20 | 4.73 | 15.35 | 12.22 | 9.79 |
|  | SD | 0.39 | 0.24 | 0.26 | 0.42 | 0.48 | 0.21 | 0.17 | 0.25 | 0.37 | 0.26 | 0.23 |
|  | Mn | 23.15 | 12.38 | 9.71 | 20.40 | 8.69 | 4.74 | 10.92 | 4.48 | 14.91 | 11.91 | 9.42 |
|  | Mx | 24.30 | 13.14 | 10.35 | 21.70 | 10.09 | 5.26 | 11.53 | 5.35 | 16.05 | 12.65 | 10.13 |
|  | CV | **1.61** | **1.92** | **2.59** | **2.01** | **5.00** | **4.26** | **1.52** | **5.32** | **2.40** | **2.13** | **2.35** |
|  | N | 10 | 9 | 10 | 10 | 6 | 6 | 9 | 10 | 9 | 10 | 10 |
| *T. hilli* | X | 19.98 | 11.09 | 7.11 | 17.18 | 8.82 | 3.56 | 8.89 | 5.33 | 13.14 | 10.85 | 6.94 |
|  | SD | 0.39 | 0.25 | 0.41 | 0.30 | 0.74 | 0.20 | 0.31 | 0.26 | 0.49 | 0.47 | 0.64 |
|  | Mn | 19.17 | 10.78 | 6.75 | 16.81 | 8.05 | 3.32 | 8.29 | 4.84 | 12.20 | 9.99 | 6.21 |
|  | Mx | 20.31 | 11.41 | 7.94 | 17.73 | 10.16 | 3.81 | 9.18 | 5.59 | 13.65 | 11.43 | 8.26 |
|  | CV | **1.96** | **2.29** | **5.75** | **1.76** | **8.37** | **5.66** | **3.50** | **4.95** | **3.72** | **4.37** | **9.17** |
|  | N | 7 | 7 | 7 | 7 | 6 | 6 | 7 | 7 | 7 | 7 | 7 |
| *T. australis* | X | 21.91 | 11.94 | 9.16 | 18.48 | 10.96 | 4.28 | 9.73 | 5.12 | 13.91 | 11.44 | 7.57 |
|  | SD | 0.43 | 0.23 | 0.42 | 1.26 | 0.35 | 0.26 | 0.61 | 0.19 | 0.83 | 0.56 | 0.33 |
|  | Mn | 21.09 | 11.43 | 8.50 | 15.39 | 10.48 | 3.95 | 8.40 | 4.70 | 12.48 | 10.16 | 7.14 |
|  | Mx | 22.50 | 12.22 | 9.68 | 19.17 | 11.43 | 4.76 | 10.24 | 5.24 | 15.08 | 11.90 | 7.94 |
|  | CV | **1.98** | **1.97** | **4.55** | **6.82** | **3.20** | **5.96** | **6.28** | **3.76** | **5.97** | **4.85** | **4.36** |
|  | N | 7 | 8 | 9 | 8 | 8 | 9 | 8 | 9 | 9 | 9 | 9 |
| *Saccolaimus* | X | 25.42 | 14.80 | 11.06 | 22.13 | 11.21 | 6.97 | 12.38 | 5.95 | 17.13 | 13.07 | 10.72 |
| *flaviventris* | SD | 0.62 | 0.86 | 0.68 | 0.69 | 1.06 | 0.10 | 0.24 | 0.20 | 0.73 | 0.93 | 0.72 |
|  | Mn | 24.80 | 13.65 | 10.16 | 21.50 | 9.35 | 6.83 | 12.14 | 5.71 | 16.51 | 11.75 | 9.85 |
|  | Mx | 26.15 | 15.87 | 12.06 | 23.18 | 12.06 | 7.15 | 12.70 | 6.35 | 18.25 | 14.29 | 11.90 |
|  | CV | **2.43** | **5.79** | **6.18** | **3.12** | **9.42** | **1.42** | **1.92** | **3.41** | **4.25** | **7.11** | **6.70** |
|  | N | 6 | 6 | 7 | 6 | 6 | 7 | 7 | 7 | 6 | 6 | 7 |

| **Species** | **Parameters** | **ANS** | **PNS** | **CH** | **DL** | **C1-M3** | **RC** | **ICD** | **P4-M3** | **M1-M3** |
| --- | --- | --- | --- | --- | --- | --- | --- | --- | --- | --- |
| *T.georgianus* | X | 9.37 | 6.57 | 7.50 | 18.38 | 11.39 | 6.24 | 3.24 | 6.76 | 5.37 |
| (all specimens) | SD | 0.47 | 0.33 | 0.33 | 0.67 | 0.37 | 0.58 | 0.19 | 0.23 | 0.21 |
|  | Mn | 8.25 | 5.87 | 7.00 | 16.11 | 10.32 | 5.00 | 3.02 | 6.00 | 4.92 |
|  | Mx | 10.39 | 7.56 | 8.35 | 20.24 | 12.38 | 7.88 | 3.81 | 7.18 | 5.92 |
|  | CV | **5.04** | **5.10** | **4.43** | **3.64** | **3.25** | **9.33** | **5.75** | **3.38** | **3.85** |
|  | N | 53 | 55 | 54 | 55 | 49 | 55 | 20 | 55 | 55 |
| *Taphozous* | X | 9.22 | 6.54 | 7.36 | 18.17 | 11.27 | 6.09 | 3.18 | 6.71 | 5.31 |
| *georgianus* | SD | 0.43 | 0.30 | 0.18 | 0.51 | 0.25 | 0.54 | 0.13 | 0.20 | 0.19 |
| (specimens | Mn | 8.41 | 6.03 | 7.00 | 16.99 | 10.79 | 5.16 | 3.02 | 6.24 | 4.92 |
| West of Mt Isa | Mx | 10.11 | 7.29 | 7.66 | 19.02 | 11.90 | 7.13 | 3.35 | 7.12 | 5.72 |
| only) | CV | **4.67** | **4.54** | **2.39** | **2.79** | **2.18** | **8.89** | **3.99** | **2.93** | **3.50** |
|  | N | 33 | 34 | 34 | 34 | 32 | 34 | 14 | 34 | 34 |
| *T. troughtoni* | X | 10.28 | 7.32 | 8.15 | 20.27 | 12.63 | 7.30 | 0.00 | 7.31 | 5.78 |
|  | SD | 0.46 | 0.30 | 0.18 | 0.81 | 0.33 | 0.58 | 0.00 | 0.18 | 0.17 |
|  | Mn | 9.42 | 6.71 | 7.90 | 19.20 | 12.13 | 6.49 | 0.00 | 7.04 | 5.49 |
|  | Mx | 10.80 | 7.86 | 8.45 | 21.47 | 13.14 | 8.04 | 0.00 | 7.59 | 6.02 |
|  | CV | **4.48** | **4.12** | **2.19** | **3.98** | **2.60** | **7.97** | **?** | **2.51** | **2.86** |
|  | N | 10 | 10 | 10 | 10 | 8 | 8 | 0 | 10 | 10 |
| *T. hilli* | X | 8.86 | 6.44 | 7.47 | 16.32 | 9.96 | 5.52 | 2.68 | 5.87 | 4.80 |
|  | SD | 0.44 | 0.14 | 0.16 | 0.20 | 0.21 | 0.21 | 0.16 | 0.09 | 0.10 |
|  | Mn | 8.01 | 6.21 | 7.30 | 15.99 | 9.70 | 5.16 | 2.42 | 5.76 | 4.69 |
|  | Mx | 9.31 | 6.59 | 7.70 | 16.59 | 10.24 | 5.75 | 2.82 | 6.04 | 4.96 |
|  | CV | **5.02** | **2.17** | **2.15** | **1.22** | **2.09** | **3.79** | **5.94** | **1.61** | **1.98** |
|  | N | 7 | 7 | 6 | 7 | 7 | 7 | 5 | 7 | 7 |
| *T. australis* | X | 8.72 | 6.28 | 7.71 | 17.48 | 10.87 | 5.60 | 3.27 | 6.38 | 5.11 |
|  | SD | 0.45 | 0.74 | 0.22 | 0.78 | 0.50 | 0.50 | 0.30 | 0.25 | 0.22 |
|  | Mn | 7.94 | 4.40 | 7.28 | 16.02 | 10.25 | 4.92 | 2.96 | 5.96 | 4.72 |
|  | Mx | 9.21 | 6.83 | 8.03 | 18.33 | 11.43 | 6.27 | 3.81 | 6.68 | 5.32 |
|  | CV | **5.20** | **11.84** | **2.87** | **4.47** | **4.57** | **9.02** | **9.14** | **3.98** | **4.25** |
|  | N | 9 | 9 | 8 | 7 | 8 | 8 | 7 | 9 | 9 |
| *S. flaviventris* | X | 11.26 | 9.95 | 8.62 | 22.12 | 14.01 | 7.35 | 4.44 | 8.03 | 6.45 |
|  | SD | 0.21 | 0.34 | 0.33 | 0.37 | 0.28 | 0.36 | 0.23 | 0.23 | 0.25 |
|  | Mn | 10.95 | 9.37 | 8.20 | 21.83 | 13.58 | 6.67 | 4.13 | 7.71 | 6.12 |
|  | Mx | 11.53 | 10.32 | 8.93 | 22.86 | 14.44 | 7.77 | 4.76 | 8.32 | 6.77 |
|  | CV | **1.89** | **3.46** | **3.81** | **1.67** | **2.00** | **4.89** | **5.18** | **2.87** | **3.93** |
|  | N | 7 | 7 | 6 | 7 | 7 | 7 | 6 | 7 | 7 |

| **Species** | **Parameters** | **C1 L** | **P4 L** | **M1 L** | **M2 L** | **M3 L** | **C1 W** | **P4 W** | **M1 BUCL** | **M1 LINL** | **M1 W** | **M1 PCH** |
| --- | --- | --- | --- | --- | --- | --- | --- | --- | --- | --- | --- | --- |
| *T.georgianus* | X | 2.27 | 1.71 | 2.44 | 2.26 | 1.04 | 1.40 | 1.47 | 2.42 | 2.37 | 1.98 | 1.99 |
|  | SD | 0.15 | 0.07 | 0.11 | 0.09 | 0.06 | 0.12 | 0.11 | 0.11 | 0.11 | 0.15 | 0.13 |
|  | Mn | 1.94 | 1.56 | 2.18 | 1.96 | 0.88 | 1.16 | 1.22 | 2.18 | 2.08 | 1.66 | 1.72 |
|  | Mx | 2.65 | 1.92 | 2.72 | 2.42 | 1.20 | 1.76 | 1.70 | 2.72 | 2.60 | 2.42 | 2.34 |
|  | CV | **6.52** | **3.84** | **4.56** | **4.10** | **6.20** | **8.45** | **7.26** | **4.65** | **4.72** | **7.62** | **6.48** |
|  | N | 53 | 55 | 55 | 54 | 55 | 52 | 55 | 55 | 55 | 55 | 55 |
| *T. georgianus* | X | 2.21 | 1.71 | 2.42 | 2.24 | 1.04 | 1.38 | 1.46 | 2.40 | 2.33 | 1.98 | 1.97 |
| (specimens from | SD | 0.09 | 0.07 | 0.11 | 0.09 | 0.06 | 0.11 | 0.10 | 0.11 | 0.11 | 0.14 | 0.13 |
| West of Mt Isa | Mn | 2.02 | 1.58 | 2.20 | 2.06 | 0.90 | 1.20 | 1.30 | 2.18 | 2.08 | 1.67 | 1.73 |
| Only) | Mx | 2.39 | 1.92 | 2.72 | 2.40 | 1.20 | 1.68 | 1.63 | 2.72 | 2.60 | 2.30 | 2.24 |
|  | CV | **4.17** | **4.00** | **4.60** | **3.82** | **6.14** | **7.99** | **6.65** | **4.65** | **4.70** | **7.14** | **6.58** |
|  | N | 33 | 34 | 34 | 34 | 34 | 32 | 34 | 34 | 34 | 34 | 34 |
| *T. troughtoni* | X | 2.56 | 1.86 | 2.60 | 2.45 | 1.18 | 1.47 | 1.54 | 2.57 | 2.53 | 2.16 | 2.13 |
|  | SD | 0.10 | 0.06 | 0.12 | 0.11 | 0.07 | 0.07 | 0.07 | 0.14 | 0.10 | 0.10 | 0.08 |
|  | Mn | 2.37 | 1.75 | 2.39 | 2.30 | 1.11 | 1.38 | 1.44 | 2.40 | 2.39 | 1.98 | 1.95 |
|  | Mx | 2.70 | 1.95 | 2.86 | 2.65 | 1.34 | 1.59 | 1.64 | 2.90 | 2.71 | 2.29 | 2.21 |
|  | CV | **3.86** | **3.08** | **4.73** | **4.49** | **5.73** | **4.60** | **4.61** | **5.54** | **4.15** | **4.83** | **3.53** |
|  | N | 9 | 10 | 10 | 10 | 10 | 9 | 10 | 10 | 10 | 10 | 9 |
| *T. hilli* | X | 1.97 | 1.43 | 2.11 | 2.02 | 0.99 | 1.05 | 1.34 | 2.09 | 2.06 | 1.76 | 1.72 |
|  | SD | 0.09 | 0.10 | 0.06 | 0.07 | 0.04 | 0.06 | 0.18 | 0.07 | 0.08 | 0.21 | 0.12 |
|  | Mn | 1.90 | 1.33 | 2.03 | 1.89 | 0.90 | 0.96 | 1.16 | 2.02 | 1.93 | 1.61 | 1.53 |
|  | Mx | 2.12 | 1.62 | 2.22 | 2.12 | 1.04 | 1.16 | 1.70 | 2.22 | 2.14 | 2.22 | 1.86 |
|  | CV | **4.52** | **7.23** | **3.02** | **3.55** | **4.53** | **5.73** | **13.57** | **3.11** | **3.92** | **12.01** | **7.02** |
|  | N | 7 | 7 | 7 | 7 | 7 | 7 | 7 | 7 | 7 | 7 | 7 |
| *T. australis* | X | 2.28 | 1.69 | 2.23 | 2.12 | 1.00 | 1.20 | 1.43 | 2.22 | 2.13 | 2.06 | 1.94 |
|  | SD | 0.14 | 0.13 | 0.09 | 0.08 | 0.07 | 0.05 | 0.09 | 0.09 | 0.15 | 0.11 | 0.15 |
|  | Mn | 2.00 | 1.40 | 2.06 | 1.98 | 0.90 | 1.10 | 1.26 | 2.04 | 1.88 | 1.90 | 1.66 |
|  | Mx | 2.46 | 1.82 | 2.34 | 2.22 | 1.08 | 1.24 | 1.56 | 2.34 | 2.38 | 2.16 | 2.22 |
|  | CV | **6.17** | **7.71** | **3.94** | **3.63** | **6.58** | **4.04** | **6.06** | **4.23** | **7.14** | **5.15** | **7.83** |
|  | N | 9 | 9 | 9 | 9 | 9 | 9 | 9 | 9 | 9 | 9 | 9 |
| *S. flaviventris* | X | 2.43 | 1.91 | 3.10 | 2.81 | 1.14 | 2.00 | 2.12 | 3.05 | 2.82 | 2.78 | 2.53 |
|  | SD | 0.17 | 0.15 | 0.13 | 0.11 | 0.07 | 0.15 | 0.09 | 0.12 | 0.19 | 0.29 | 0.21 |
|  | Mn | 2.20 | 1.68 | 2.89 | 2.64 | 1.02 | 1.84 | 2.04 | 2.92 | 2.48 | 2.32 | 2.24 |
|  | Mx | 2.70 | 2.06 | 3.26 | 2.90 | 1.22 | 2.26 | 2.29 | 3.20 | 3.10 | 3.10 | 2.94 |
|  | CV | **7.16** | **7.70** | **4.34** | **3.84** | **6.02** | **7.64** | **4.06** | **4.00** | **6.88** | **10.54** | **8.47** |
|  | N | 7 | 7 | 7 | 7 | 7 | 7 | 7 | 7 | 7 | 7 | 7 |

| **Species** | **Parameters** | **M1 PMC** | **M1 PPC** | **M1 HW** | **M2**  **BUC L** | **M2**  **LIN L** | **M2 W** | **M2 PCH** | **M2**  **PMC** | **M2 PPC** | **M2 HW** | **M3 BUC L** |
| --- | --- | --- | --- | --- | --- | --- | --- | --- | --- | --- | --- | --- |
| *T.georgianus* | X | 1.64 | 1.02 | 1.08 | 2.25 | 2.09 | 2.17 | 1.72 | 1.56 | 1.11 | 1.14 | 0.93 |
|  | SD | 0.09 | 0.07 | 0.08 | 0.12 | 0.11 | 0.12 | 0.12 | 0.09 | 0.07 | 0.09 | 0.15 |
|  | Mn | 1.37 | 0.83 | 0.91 | 1.74 | 1.80 | 1.90 | 1.50 | 1.25 | 0.96 | 0.95 | 0.48 |
|  | Mx | 1.85 | 1.16 | 1.26 | 2.46 | 2.31 | 2.60 | 1.97 | 1.71 | 1.26 | 1.42 | 1.16 |
|  | CV | **5.61** | **6.51** | **7.28** | **5.13** | **5.21** | **5.34** | **6.79** | **5.96** | **5.98** | **8.01** | **16.47** |
|  | N | 55 | 55 | 54 | 54 | 54 | 54 | 54 | 54 | 54 | 54 | 55 |
| *T. georgianus* | X | 1.63 | 1.02 | 1.07 | 2.25 | 2.06 | 2.17 | 1.70 | 1.55 | 1.11 | 1.13 | 0.89 |
| (specimens from | SD | 0.09 | 0.07 | 0.07 | 0.08 | 0.09 | 0.10 | 0.11 | 0.08 | 0.07 | 0.09 | 0.16 |
| West of Mt Isa | Mn | 1.42 | 0.83 | 0.91 | 2.13 | 1.90 | 1.98 | 1.50 | 1.36 | 0.96 | 0.95 | 0.48 |
| Only) | Mx | 1.85 | 1.15 | 1.22 | 2.40 | 2.24 | 2.38 | 1.95 | 1.71 | 1.26 | 1.42 | 1.16 |
|  | CV | **5.76** | **6.80** | **6.84** | **3.71** | **4.37** | **4.46** | **6.24** | **5.42** | **6.50** | **7.89** | **17.82** |
|  | N | 34 | 34 | 34 | 34 | 34 | 34 | 34 | 34 | 34 | 34 | 34 |
| *T. troughtoni* | X | 1.72 | 1.12 | 1.19 | 2.46 | 2.27 | 2.37 | 1.89 | 1.69 | 1.21 | 1.25 | 1.17 |
|  | SD | 0.07 | 0.06 | 0.06 | 0.10 | 0.07 | 0.16 | 0.12 | 0.08 | 0.07 | 0.04 | 0.07 |
|  | Mn | 1.64 | 1.03 | 1.10 | 2.24 | 2.17 | 2.01 | 1.63 | 1.54 | 1.12 | 1.18 | 1.11 |
|  | Mx | 1.87 | 1.21 | 1.28 | 2.59 | 2.39 | 2.55 | 2.01 | 1.83 | 1.35 | 1.33 | 1.35 |
|  | CV | **4.21** | **5.11** | **5.30** | **4.18** | **3.05** | **6.61** | **6.55** | **4.68** | **5.96** | **3.52** | **6.15** |
|  | N | 9 | 9 | 10 | 10 | 10 | 10 | 9 | 10 | 9 | 9 | 10 |
| *T. hilli* | X | 1.42 | 0.97 | 0.98 | 2.02 | 1.86 | 1.89 | 1.62 | 1.42 | 1.09 | 1.01 | 0.97 |
|  | SD | 0.09 | 0.05 | 0.07 | 0.08 | 0.09 | 0.19 | 0.19 | 0.06 | 0.07 | 0.12 | 0.14 |
|  | Mn | 1.27 | 0.92 | 0.89 | 1.89 | 1.74 | 1.74 | 1.36 | 1.35 | 1.01 | 0.85 | 0.67 |
|  | Mx | 1.51 | 1.05 | 1.10 | 2.14 | 1.98 | 2.28 | 1.96 | 1.52 | 1.20 | 1.20 | 1.05 |
|  | CV | **6.14** | **4.78** | **6.94** | **3.92** | **4.63** | **9.92** | **11.88** | **4.45** | **6.72** | **11.90** | **14.35** |
|  | N | 7 | 7 | 7 | 7 | 7 | 7 | 7 | 7 | 7 | 7 | 7 |
| *T. australis* | X | 1.36 | 0.98 | 1.03 | 2.12 | 2.03 | 2.18 | 1.75 | 1.34 | 1.07 | 1.07 | 0.75 |
|  | SD | 0.07 | 0.04 | 0.06 | 0.08 | 0.10 | 0.09 | 0.09 | 0.07 | 0.04 | 0.06 | 0.10 |
|  | Mn | 1.23 | 0.92 | 0.95 | 2.02 | 1.88 | 2.00 | 1.56 | 1.26 | 1.00 | 1.00 | 0.54 |
|  | Mx | 1.46 | 1.02 | 1.10 | 2.22 | 2.14 | 2.28 | 1.86 | 1.48 | 1.12 | 1.20 | 0.90 |
|  | CV | **4.89** | **3.63** | **5.53** | **3.59** | **4.83** | **4.09** | **5.05** | **5.47** | **3.92** | **5.80** | **12.68** |
|  | N | 9 | 9 | 9 | 9 | 9 | 9 | 9 | 9 | 9 | 9 | 9 |
| *S. flaviventris* | X | 2.17 | 1.26 | 1.35 | 2.76 | 2.63 | 2.85 | 2.25 | 2.00 | 1.25 | 1.42 | 0.93 |
|  | SD | 0.13 | 0.06 | 0.17 | 0.11 | 0.18 | 0.18 | 0.16 | 0.10 | 0.10 | 0.12 | 0.16 |
|  | Mn | 2.01 | 1.20 | 1.10 | 2.62 | 2.43 | 2.60 | 2.04 | 1.80 | 1.13 | 1.18 | 0.77 |
|  | Mx | 2.34 | 1.35 | 1.55 | 2.91 | 2.88 | 3.08 | 2.46 | 2.10 | 1.42 | 1.58 | 1.23 |
|  | CV | **5.77** | **5.05** | **12.95** | **3.80** | **6.75** | **6.31** | **6.95** | **5.08** | **7.76** | **8.58** | **17.50** |
|  | N | 7 | 7 | 7 | 7 | 7 | 7 | 7 | 7 | 7 | 7 | 7 |

| **Species** | **Parameters** | **M3 LIN L** | **M3 W** | **P4-M3** | **M1-M3** | **DEN D C1** | **DEN D**  **P4** | **L C1** | **L P4** | **L M1** | **L M2** | **L M3** |
| --- | --- | --- | --- | --- | --- | --- | --- | --- | --- | --- | --- | --- |
| *T.georgianus* | X | 0.79 | 1.72 | 8.16 | 6.55 | 1.69 | 2.38 | 1.65 | 1.75 | 2.38 | 2.36 | 1.97 |
|  | SD | 0.17 | 0.26 | 0.32 | 0.26 | 0.17 | 0.17 | 0.11 | 0.08 | 0.12 | 0.10 | 0.09 |
|  | Mn | 0.40 | 0.91 | 7.24 | 5.80 | 1.25 | 2.14 | 1.29 | 1.52 | 2.04 | 2.14 | 1.70 |
|  | Mx | 1.07 | 2.08 | 9.04 | 7.20 | 2.16 | 2.81 | 1.84 | 1.94 | 2.58 | 2.64 | 2.18 |
|  | CV | **21.15** | **15.07** | **3.92** | **4.04** | **10.14** | **6.99** | **6.44** | **4.55** | **5.12** | **4.34** | **4.51** |
|  | N | 50 | 55 | 53 | 53 | 47 | 49 | 49 | 53 | 53 | 55 | 55 |
| *T. georgianus* | X | 0.76 | 1.65 | 8.03 | 6.43 | 1.64 | 2.37 | 1.62 | 1.73 | 2.34 | 2.34 | 1.96 |
| (specimens from | SD | 0.18 | 0.27 | 0.22 | 0.18 | 0.17 | 0.16 | 0.10 | 0.06 | 0.10 | 0.10 | 0.08 |
| West of Mt Isa | Mn | 0.40 | 0.91 | 7.52 | 6.00 | 1.25 | 2.14 | 1.29 | 1.59 | 2.16 | 2.14 | 1.79 |
| Only) | Mx | 1.07 | 2.08 | 8.49 | 6.80 | 1.99 | 2.67 | 1.80 | 1.84 | 2.58 | 2.64 | 2.18 |
|  | CV | **23.14** | **16.61** | **2.74** | **2.72** | **10.39** | **6.92** | **6.10** | **3.52** | **4.42** | **4.20** | **4.26** |
|  | N | 32 | 34 | 34 | 33 | 31 | 32 | 32 | 34 | 33 | 34 | 34 |
| *T. troughtoni* | X | 1.02 | 2.06 | 9.22 | 7.14 | 1.92 | 2.60 | 1.91 | 1.91 | 2.59 | 2.52 | 2.19 |
|  | SD | 0.11 | 0.17 | 0.67 | 0.26 | 0.18 | 0.19 | 0.09 | 0.02 | 0.10 | 0.12 | 0.08 |
|  | Mn | 0.89 | 1.82 | 8.48 | 6.82 | 1.72 | 2.25 | 1.74 | 1.88 | 2.46 | 2.39 | 2.09 |
|  | Mx | 1.22 | 2.37 | 10.70 | 7.61 | 2.18 | 2.78 | 2.03 | 1.95 | 2.73 | 2.73 | 2.31 |
|  | CV | **10.53** | **8.31** | **7.24** | **3.58** | **9.34** | **7.39** | **4.47** | **1.30** | **3.69** | **4.82** | **3.72** |
|  | N | 9 | 10 | 8 | 7 | 7 | 7 | 9 | 8 | 7 | 9 | 8 |
| *T. hilli* | X | 0.87 | 1.61 | 7.01 | 5.71 | 1.50 | 2.31 | 1.30 | 1.44 | 2.10 | 2.06 | 1.77 |
|  | SD | 0.17 | 0.13 | 0.19 | 0.19 | 0.09 | 0.16 | 0.05 | 0.09 | 0.07 | 0.07 | 0.09 |
|  | Mn | 0.58 | 1.41 | 6.77 | 5.38 | 1.38 | 2.08 | 1.24 | 1.34 | 1.96 | 1.94 | 1.65 |
|  | Mx | 1.06 | 1.76 | 7.28 | 5.92 | 1.68 | 2.52 | 1.40 | 1.60 | 2.16 | 2.16 | 1.90 |
|  | CV | **19.86** | **8.25** | **2.76** | **3.33** | **6.32** | **7.01** | **4.11** | **6.26** | **3.37** | **3.38** | **5.12** |
|  | N | 6 | 7 | 7 | 7 | 7 | 7 | 7 | 7 | 7 | 7 | 7 |
| *T. australis* | X | 0.59 | 1.48 | 7.72 | 6.23 | 1.43 | 2.23 | 1.53 | 1.71 | 2.20 | 2.14 | 1.91 |
|  | SD | 0.08 | 0.16 | 0.26 | 0.32 | 0.17 | 0.27 | 0.08 | 0.12 | 0.05 | 0.06 | 0.06 |
|  | Mn | 0.42 | 1.08 | 7.20 | 5.76 | 1.08 | 1.66 | 1.38 | 1.44 | 2.10 | 2.02 | 1.84 |
|  | Mx | 0.71 | 1.60 | 8.16 | 6.88 | 1.60 | 2.40 | 1.64 | 1.84 | 2.26 | 2.22 | 2.06 |
|  | CV | **12.84** | **11.00** | **3.41** | **5.19** | **11.91** | **11.96** | **5.32** | **6.98** | **2.26** | **2.92** | **3.40** |
|  | N | 9 | 9 | 9 | 9 | 7 | 7 | 9 | 9 | 9 | 9 | 9 |
| *S. flaviventris* | X | 0.68 | 1.79 | 9.80 | 8.05 | 2.69 | 2.59 | 2.22 | 2.03 | 2.98 | 2.97 | 2.43 |
|  | SD | 0.28 | 0.26 | 0.35 | 0.32 | 0.14 | 0.38 | 0.15 | 0.10 | 0.11 | 0.10 | 0.10 |
|  | Mn | 0.44 | 1.55 | 9.44 | 7.72 | 2.50 | 2.18 | 1.97 | 1.91 | 2.86 | 2.85 | 2.26 |
|  | Mx | 0.99 | 2.28 | 10.54 | 8.52 | 2.84 | 3.20 | 2.40 | 2.14 | 3.14 | 3.08 | 2.54 |
|  | CV | **41.41** | **14.26** | **3.61** | **3.96** | **5.24** | **14.72** | **6.93** | **4.75** | **3.65** | **3.32** | **4.27** |
|  | N | 3 | 6 | 7 | 7 | 5 | 5 | 7 | 7 | 7 | 7 | 6 |

| **Species** | **Parameters** | **W C1** | **W P4** | **TRIL M1** | **TALL M1** | **TRIW M1** | **TALW**  **M1** | **PARA M1** | **META M1** | **TRIL M2** | **TALL M2** | **TRIW M2** |
| --- | --- | --- | --- | --- | --- | --- | --- | --- | --- | --- | --- | --- |
| *T.georgianus* | X | 1.15 | 1.03 | 1.13 | 1.25 | 1.32 | 1.51 | 1.01 | 0.81 | 1.02 | 1.33 | 1.47 |
|  | SD | 0.09 | 0.08 | 0.08 | 0.08 | 0.10 | 0.09 | 0.12 | 0.15 | 0.07 | 0.09 | 0.11 |
|  | Mn | 0.94 | 0.88 | 0.96 | 1.06 | 1.14 | 1.32 | 0.70 | 0.57 | 0.90 | 0.96 | 1.24 |
|  | Mx | 1.30 | 1.38 | 1.30 | 1.44 | 1.54 | 1.68 | 1.24 | 1.44 | 1.20 | 1.48 | 1.68 |
|  | CV | **7.96** | **8.08** | **6.80** | **6.62** | **7.47** | **6.05** | **11.90** | **18.19** | **6.77** | **6.75** | **7.43** |
|  | N | 49 | 54 | 54 | 54 | 54 | 54 | 54 | 54 | 55 | 55 | 55 |
| *T. georgianus* | X | 1.11 | 1.01 | 1.11 | 1.23 | 1.26 | 1.47 | 1.03 | 0.80 | 1.01 | 1.33 | 1.43 |
| (specimens from | SD | 0.08 | 0.08 | 0.07 | 0.08 | 0.07 | 0.07 | 0.11 | 0.11 | 0.06 | 0.07 | 0.08 |
| West of Mt Isa | Mn | 0.94 | 0.88 | 0.96 | 1.06 | 1.14 | 1.32 | 0.82 | 0.60 | 0.90 | 1.18 | 1.29 |
| Only) | Mx | 1.29 | 1.38 | 1.30 | 1.36 | 1.42 | 1.63 | 1.24 | 1.00 | 1.16 | 1.48 | 1.63 |
|  | CV | **6.87** | **8.39** | **6.72** | **6.33** | **5.72** | **4.87** | **10.26** | **13.84** | **6.27** | **5.62** | **5.75** |
|  | N | 32 | 34 | 33 | 33 | 33 | 33 | 33 | 33 | 34 | 34 | 34 |
| *T. troughtoni* | X | 1.25 | 1.15 | 1.21 | 1.31 | 1.52 | 1.68 | 0.99 | 0.79 | 1.18 | 1.30 | 1.60 |
|  | SD | 0.04 | 0.05 | 0.09 | 0.12 | 0.08 | 0.08 | 0.06 | 0.05 | 0.08 | 0.13 | 0.10 |
|  | Mn | 1.21 | 1.08 | 1.12 | 1.10 | 1.44 | 1.57 | 0.93 | 0.71 | 1.09 | 1.07 | 1.42 |
|  | Mx | 1.31 | 1.23 | 1.37 | 1.42 | 1.65 | 1.82 | 1.11 | 0.86 | 1.31 | 1.44 | 1.71 |
|  | CV | 3.09 | **3.98** | **7.09** | **9.08** | **5.37** | **4.96** | **6.51** | **6.68** | **6.45** | **9.99** | **6.15** |
|  | N | 9 | 8 | 7 | 7 | 7 | 7 | 7 | 7 | 9 | 9 | 9 |
| *T. hilli* | X | 0.99 | 0.85 | 1.00 | 1.10 | 1.10 | 1.31 | 0.92 | 0.74 | 0.85 | 1.20 | 1.25 |
|  | SD | 0.04 | 0.05 | 0.04 | 0.08 | 0.04 | 0.06 | 0.12 | 0.14 | 0.07 | 0.09 | 0.06 |
|  | Mn | 0.93 | 0.81 | 0.94 | 0.95 | 1.03 | 1.19 | 0.78 | 0.56 | 0.79 | 1.04 | 1.16 |
|  | Mx | 1.03 | 0.94 | 1.07 | 1.21 | 1.16 | 1.40 | 1.13 | 0.96 | 0.98 | 1.30 | 1.35 |
|  | CV | **4.01** | **5.91** | **4.31** | **7.44** | **4.04** | **4.96** | **13.10** | **18.72** | **8.25** | **7.34** | **4.51** |
|  | N | 7 | 7 | 7 | 7 | 7 | 7 | 7 | 7 | 7 | 7 | 7 |
| *T. australis* | X | 1.07 | 0.95 | 1.11 | 1.08 | 1.14 | 1.30 | 1.12 | 0.78 | 0.97 | 1.14 | 1.23 |
|  | SD | 0.10 | 0.15 | 0.05 | 0.06 | 0.16 | 0.07 | 0.08 | 0.06 | 0.08 | 0.07 | 0.06 |
|  | Mn | 0.96 | 0.76 | 1.02 | 0.98 | 0.94 | 1.16 | 1.01 | 0.70 | 0.82 | 1.06 | 1.14 |
|  | Mx | 1.28 | 1.32 | 1.18 | 1.16 | 1.48 | 1.38 | 1.24 | 0.90 | 1.08 | 1.24 | 1.28 |
|  | CV | **9.82** | **16.26** | **4.35** | **5.28** | **13.62** | **5.18** | **7.17** | **7.48** | **7.92** | **5.77** | **4.61** |
|  | N | 9 | 9 | 9 | 9 | 9 | 9 | 9 | 9 | 9 | 9 | 9 |
| *S. flaviventris* | X | 1.56 | 1.45 | 1.44 | 1.54 | 1.78 | 1.92 | 1.28 | 1.11 | 1.36 | 1.63 | 1.82 |
|  | SD | 0.13 | 0.06 | 0.09 | 0.11 | 0.09 | 0.05 | 0.21 | 0.15 | 0.04 | 0.07 | 0.09 |
|  | Mn | 1.34 | 1.38 | 1.34 | 1.38 | 1.66 | 1.88 | 1.02 | 0.90 | 1.30 | 1.53 | 1.70 |
|  | Mx | 1.74 | 1.52 | 1.56 | 1.66 | 1.90 | 2.00 | 1.62 | 1.34 | 1.42 | 1.72 | 1.92 |
|  | CV | **8.02** | **4.11** | **6.13** | **7.18** | **5.14** | **2.37** | **16.73** | **13.73** | **2.87** | **4.42** | **4.90** |
|  | N | 7 | 7 | 7 | 7 | 7 | 7 | 7 | 7 | 7 | 7 | 7 |

| **Species** | **Parameters** | **TALW M2** | **PARA M2** | **META M2** | **TRIL M3** | **TALL M3** | **TRIW M3** | **TALW M3** |
| --- | --- | --- | --- | --- | --- | --- | --- | --- |
| *T.georgianus* | X | 1.55 | 1.07 | 0.95 | 0.86 | 1.13 | 1.33 | 0.84 |
|  | SD | 0.12 | 0.11 | 0.10 | 0.06 | 0.07 | 0.11 | 0.09 |
|  | Mn | 1.30 | 0.84 | 0.75 | 0.70 | 0.94 | 1.10 | 0.68 |
|  | Mx | 1.79 | 1.30 | 1.18 | 1.00 | 1.30 | 1.53 | 1.01 |
|  | CV | **7.45** | **10.29** | **10.50** | **7.44** | **6.59** | 7.98 | **11.02** |
|  | N | 55 | 55 | 55 | 55 | 54 | 55 | 54 |
| *T. georgianus* | X | 1.52 | 1.08 | 0.95 | 0.85 | 1.13 | 1.29 | 0.81 |
| (specimens from | SD | 0.11 | 0.11 | 0.10 | 0.07 | 0.07 | 0.09 | 0.09 |
| West of Mt Isa | Mn | 1.30 | 0.84 | 0.75 | 0.70 | 1.01 | 1.10 | 0.68 |
| Only) | Mx | 1.79 | 1.26 | 1.18 | 1.00 | 1.30 | 1.48 | 1.01 |
|  | CV | **7.22** | **9.84** | **10.68** | **8.24** | **6.41** | **6.99** | **11.43** |
|  | N | 34 | 34 | 34 | 34 | 33 | 34 | 33 |
| *T. troughtoni* | X | 1.71 | 1.11 | 0.98 | 0.94 | 1.27 | 1.50 | 0.98 |
|  | SD | 0.06 | 0.13 | 0.09 | 0.08 | 0.11 | 0.09 | 0.07 |
|  | Mn | 1.61 | 0.99 | 0.89 | 0.81 | 1.09 | 1.36 | 0.89 |
|  | Mx | 1.79 | 1.43 | 1.19 | 1.07 | 1.42 | 1.61 | 1.08 |
|  | CV | **3.73** | **12.14** | **9.62** | **8.49** | **8.56** | **5.67** | **6.86** |
|  | N | 9 | 9 | 9 | 8 | 8 | 8 | 8 |
| *T. hilli* | X | 1.35 | 0.99 | 0.86 | 0.73 | 1.03 | 1.16 | 0.77 |
|  | SD | 0.06 | 0.11 | 0.11 | 0.06 | 0.08 | 0.05 | 0.05 |
|  | Mn | 1.26 | 0.77 | 0.71 | 0.66 | 0.89 | 1.08 | 0.70 |
|  | Mx | 1.41 | 1.10 | 1.01 | 0.84 | 1.13 | 1.24 | 0.87 |
|  | CV | **4.17** | **11.52** | **13.26** | **7.64** | **7.54** | **4.23** | **6.78** |
|  | N | 7 | 7 | 7 | 7 | 7 | 7 | 7 |
| *T. australis* | X | 1.33 | 1.15 | 1.03 | 0.83 | 1.07 | 1.15 | 0.77 |
|  | SD | 0.09 | 0.07 | 0.08 | 0.08 | 0.07 | 0.05 | 0.06 |
|  | Mn | 1.12 | 1.06 | 0.90 | 0.68 | 1.00 | 1.06 | 0.68 |
|  | Mx | 1.42 | 1.28 | 1.14 | 0.94 | 1.18 | 1.20 | 0.84 |
|  | CV | **6.86** | **5.80** | **7.95** | **9.64** | **6.32** | **3.91** | **7.45** |
|  | N | 9 | 9 | 9 | 9 | 9 | 9 | 9 |
| *S. flaviventris* | X | 1.86 | 1.38 | 1.23 | 1.16 | 1.35 | 1.62 | 1.04 |
|  | SD | 0.10 | 0.14 | 0.13 | 0.05 | 0.11 | 0.09 | 0.12 |
|  | Mn | 1.74 | 1.12 | 1.02 | 1.08 | 1.23 | 1.50 | 0.88 |
|  | Mx | 2.03 | 1.52 | 1.40 | 1.24 | 1.50 | 1.76 | 1.23 |
|  | CV | **5.44** | **10.29** | **10.82** | **4.63** | **7.78** | **5.32** | **11.99** |
|  | N | 7 | 7 | 7 | 7 | 7 | 7 | 7 |

Table S3.3**: Univariate statistics for Rackham’s Roost Site fossil emballonurid specimens. Abbreviations in Glossary, below**

| **Species** | **Parameters** | **C1 L** | **P4 L** | **M1 L** | **M2 L** | **M3 L** | **C1 W** | **P4 W** | **M1**  **BUCL** | **M1**  **LINL** | **M1 W** | **M1 PCH** |
| --- | --- | --- | --- | --- | --- | --- | --- | --- | --- | --- | --- | --- |
| *Fossil specimens* | X | 2.04 | 2.10 | 2.59 | 2.50 | 0.00 | 1.15 | 1.58 | 2.44 | 2.58 | 2.07 | 2.13 |
|  | SD | 0.15 | 0.16 | 0.17 | 0.23 | 0.00 | 0.11 | 0.08 | 0.15 | 0.17 | 0.20 | 0.20 |
|  | Mn | 1.70 | 1.92 | 2.19 | 2.13 | 0.00 | 0.98 | 1.51 | 2.11 | 2.20 | 1.69 | 1.73 |
|  | Mx | 2.23 | 2.23 | 2.80 | 3.00 | 0.00 | 1.31 | 1.66 | 2.58 | 2.80 | 2.34 | 2.39 |
|  | CV | **7.20** | **7.73** | **6.51** | **9.08** | **0.00** | **9.69** | **4.84** | **6.10** | **6.69** | **9.61** | **9.38** |
|  | N | 13 | 3 | 12 | 13 | 0 | 13 | 3 | 12 | 12 | 12 | 12 |

|  |  | **M1 PMC** | **M1**  **PPC** | **M1 HW** | **M2**  **BUC L** | **M2**  **LIN L** | **M2 W** | **M2**  **PCH** | **M2**  **PMC** | **M2**  **PPC** | **M2 HW** | **M3 BUC L** |
| --- | --- | --- | --- | --- | --- | --- | --- | --- | --- | --- | --- | --- |
| **Species** | **Parameters** |  |  |  |  |  |  |  |  |  |  |  |
| *Fossil specimens* | X | 1.62 | 1.09 | 1.04 | 2.27 | 2.57 | 2.26 | 2.15 | 1.60 | 1.10 | 1.10 | 0.00 |
|  | SD | 0.07 | 0.08 | 0.12 | 0.13 | 0.21 | 0.26 | 0.21 | 0.11 | 0.06 | 0.12 | 0.00 |
|  | Mn | 1.53 | 0.99 | 0.78 | 2.10 | 2.29 | 1.84 | 1.78 | 1.44 | 0.99 | 0.85 | 0.00 |
|  | Mx | 1.72 | 1.25 | 1.28 | 2.58 | 3.00 | 2.79 | 2.46 | 1.84 | 1.20 | 1.20 | 0.00 |
|  | CV | **4.07** | **7.04** | **11.28** | **5.91** | **8.11** | **11.56** | **9.59** | **7.17** | **5.33** | **10.57** | **0.00** |
|  | N | 11 | 12 | 12 | 12 | 9 | 9 | 8 | 12 | 13 | 8 | 0 |

|  | **M3 LIN L** | | **M3 W** | **P4-M3** | **M1-M3** | **DEN D**  **C1** | **DEN D**  **P4** | **L C1** | **L P4** | **L M1** | **L M2** | **L M3** |
| --- | --- | --- | --- | --- | --- | --- | --- | --- | --- | --- | --- | --- |
| **Species** | **Parameters** |  |  |  |  |  |  |  |  |  |  |  |
| *Fossil specimens* | X | 0.00 | 0.00 | 8.51 | 0.00 | 0.00 | 0.00 | 1.64 | 1.84 | 2.42 | 2.44 | 2.08 |
|  | SD | 0.00 | 0.00 | 0.00 | 0.00 | 0.00 | 0.00 | 0.06 | 0.03 | 0.08 | 0.10 | 0.24 |
|  | Mn | 0.00 | 0.00 | 8.51 | 0.00 | 0.00 | 0.00 | 1.59 | 1.81 | 2.33 | 2.33 | 1.76 |
|  | Mx | 0.00 | 0.00 | 8.51 | 0.00 | 0.00 | 0.00 | 1.73 | 1.87 | 2.48 | 2.65 | 2.66 |
|  | CV | **0.00** | **0.00** | **0.00** | **0.00** | **0.00** | **0.00** | **3.84** | **1.66** | **3.36** | **4.07** | **11.41** |
|  | N | 0 | 0 | 1 | 0 | 0 | 0 | 4 | 3 | 3 | 10 | 10 |
|  |  |  |  |  |  |  |  |  |  |  |  |  |
|  |  |  |  |  |  |  |  |  |  |  |  |  |

| **Species** | **Parameters** | **W C1** | **W P4** | **TRIL**  **M1** | **TALL**  **M1** | **TRIW**  **M1** | **TALW**  **M1** | **PARA**  **M1** | **META**  **M1** | **TRIL**  **M2** | **TALL**  **M2** | **TRIW M2** |
| --- | --- | --- | --- | --- | --- | --- | --- | --- | --- | --- | --- | --- |
| *Fossil specimens* | X | 1.13 | 1.06 | 1.13 | 1.30 | 1.39 | 1.57 | 1.05 | 0.87 | 1.09 | 1.34 | 1.55 |
|  | SD | 0.04 | 0.12 | 0.06 | 0.03 | 0.12 | 0.14 | 0.03 | 0.08 | 0.07 | 0.15 | 0.10 |
|  | Mn | 1.08 | 0.94 | 1.06 | 1.27 | 1.27 | 1.41 | 1.02 | 0.82 | 0.96 | 1.20 | 1.43 |
|  | Mx | 1.18 | 1.17 | 1.18 | 1.32 | 1.51 | 1.66 | 1.07 | 0.96 | 1.17 | 1.60 | 1.76 |
|  | CV | **3.94** | **10.89** | **5.42** | **1.94** | **8.63** | **8.85** | **2.74** | **8.67** | **6.81** | **11.13** | **6.36** |
|  | N | 4 | 3 | 3 | 3 | 3 | 3 | 3 | 3 | 10 | 10 | 10 |

| **Species** | **Parameters** | **TALW**  **M2** | **PARA**  **M2** | **META**  **M2** | **TRIL**  **M3** | **TALL**  **M3** | **TRIW**  **M3** | **TALW**  **M3** |
| --- | --- | --- | --- | --- | --- | --- | --- | --- |
| *Fossil specimens* | X | 1.71 | 1.08 | 0.92 | 0.96 | 1.11 | 1.40 | 0.92 |
|  | SD | 0.12 | 0.12 | 0.17 | 0.16 | 0.16 | 0.24 | 0.15 |
|  | Mn | 1.53 | 0.89 | 0.61 | 0.82 | 0.87 | 1.15 | 0.76 |
|  | Mx | 1.92 | 1.29 | 1.14 | 1.37 | 1.39 | 2.04 | 1.28 |
|  | CV | **7.01** | **11.51** | **17.94** | **16.12** | **14.08** | **17.48** | **16.05** |
|  | N | 10 | 10 | 10 | 10 | 10 | 10 | 10 |

**Glossary:**

**Cranio-dental variables**

**GL:** Greatest skull length

**MW:** Mastoid width, between mastoid processes

**PL:** Palatal length

**BL:** Basicranial length, between anterior edge of foramen magnum and anterior edge of premaxilla

**OP:** Distance outside promontorium

**RC1 – LC1:** Inter upper canine distance

**C1-M3:** Upper maxillary tooth row crown length, from anterior tip of C1 to posterior edge of M3.

**P4 – M3:** Anterior tip of P4 to posterior edge of M3

**M1 – M3:** Anterior edge of M1 to posterior edge of M3

**LOW:** Least interorbital width

**ZW:** Zygomatic width

**BW:** Braincase width

**ROL:** Rostrum length, from LOW to anterior edge of premaxilla

**ANS:** Anterior nose-shield width

**PNS:** Posterior nose-shield width

**CH:** Cranial height, from apex of skull to basisphenoid

**DL:** Dentary length

**C1 – M3:** Anterior edge of C1 to posterior edge of M3

**RC:** Posterior edge of M3 to posterior edge of dentary condyle

**DEN D C_1_:** Depth of dentary below C_1_

**DEN D P_4_:** Depth of dentary below P_4_

**ICD:** Inter-lower canine distance, from outer buccal edge of left C1 to right C_1_ *

**P4 – M3:** Anterior edge of P4 to posterior edge of M3

**M1 – M3:** Anterior edge of M1 to posterior edge of M3

**C_1_ L, C^1^ L, P_4_ L, P^4^ L:** Maximum length of tooth crown

**C_1_ W, C^1^ W, P_4_ W, P^4^ W**: Maximum width of tooth crown

**Upper molar variables**

**Mx L:** Maximum crown length

**Mx BUCL:** Buccal length: Anterior tip of parastyle to posterior tip of metastyle*

**Mx LINL:** Lingual length: Anterior tip of parastyle to posterior tip of heel*

**M^X^ W:** Greatest distance between buccal edge of crown and lingual edge

**M^X^ PCH:** Paracone to heel: Enamel tip of paracone to longest tip of heel*

**M^X^ PMC:** Postmetacrista: Anterior/ buccal tip of metastyle to tip of metacone*

**M^X^ PPC:** Postparacrista: concave point of division of mesostyle to tip of paracone*

**M^X^ HW:** Heel width: widest part of heel from lingual edge to base of metacone*

**Lower molar variables**

**Mx L:** Maximum crown length, from edge of anterior cingulum/paraconid to posterior cingulum.

**Mx W**: Greatest distance between buccal edge of crown and lingual edge

**Mx TRIL:** Trigonid length, from tip of metaconid to furthest point of either paraconid or anterior cingulum (whichever is widest)*

**Mx TALL:** Talonid length, from tip of metaconid to hypoconulid/ posterior cingulum*

**Mx TRIW:** Trigonid width, from tip of metaconid to base of protoconid/ buccal cingulum*

**Mx TALW:** Talonid width, from tip of metaconid to base hypoconid/buccal cingulum*

**Mx PARA:** Paracristid length - Lingual edge of paraconid to the tip of the protoconid*

**Mx META:** Metacristid length - Tip of protoconid to tip of the metaconid*

* – removed from multivariate analyses, as preliminary analyses identified as uninformative
